# Supplementary material for: Impact of procedural variability and study design quality on the efficacy of cell-based therapies for heart failure - a meta-analysis
Source: PLoS One. 2022 Jan 5;17(1):e0261462. doi: 10.1371/journal.pone.0261462 (PMC8730409; doi:10.1371/journal.pone.0261462)
Supplement: S1 Table — Patients undergoing LVAD implantation were excluded because most deaths were due to LVAD dysfunctions, pump thrombus, multi-system organ failure, and sepsis that are likely to be irrelevant for cell therapy applications. (DOCX) [file pone.0261462.s003.docx]

| **Inclusion criteria** | **Exclusion criteria** |
| --- | --- |
| Clinical trial publications | Reviews, preclinical experiments, case reports |
| Subjects aged at least 18 years | Young subjects of less than 18 years |
| Studies written in English | Articles written in non-English languages |
| Ischemic or non-ischemic HF:  LVEF < 50%, NYHA stage II-IV, HF symptoms | HF due to Chagas disease |
| Follow up for at least 3 months after transplantation | Patients treated with LVAD implantations |
| RCTs studying cell-based therapy | Papers that were not accessible for full text |
