# Supplementary material for: Impact of procedural variability and study design quality on the efficacy of cell-based therapies for heart failure - a meta-analysis
Source: PLoS One. 2022 Jan 5;17(1):e0261462. doi: 10.1371/journal.pone.0261462 (PMC8730409; doi:10.1371/journal.pone.0261462)
Supplement: S2 Table — (DOCX) [file pone.0261462.s004.docx]

| **Outcome** | **Measurement** | **Data type** | **Data unit** | **Data source** | **Effect measurement** |
| --- | --- | --- | --- | --- | --- |
| **Primary outcomes** | | | | | |
| LVEF | Myocardial contractibility | Continuous | % | Change from baseline | Mean difference |
| Mortality | Safety | Dichotomous | Death/total patient number | Incidence at endpoint | Risk ratio |
| **Secondary outcomes** | | | | | |
| 6MW distance  Walking speed | Exercise tolerance | Continuous | meters  mph | Change from baseline | Standardized mean difference |
| BNP  NT-proBNP | HF biomarkers | Continuous | pg/ml | Change from baseline | Standardized mean difference |
| HF-MACEs | Safety | Dichotomous | Events/total patient number | Incidence at endpoint | Risk ratio |
| LVEDV  LVEDD  LVEDVI | Progression in ventricular remodeling | Continuous | ml  mm  ml/m^2^ | Change from baseline | Standardized mean difference |
| NYHA | HF symptoms | Continuous | - | Mean at endpoint | Mean difference |
| Quality of life | Score of life quality questionnaires | Continuous | - | Mean at endpoint | Mean difference |
| SAEs | Safety | Dichotomous | Events/total patient number | Incidence at endpoint | Risk ratio |
