# Supplementary material for: Impact of procedural variability and study design quality on the efficacy of cell-based therapies for heart failure - a meta-analysis
Source: PLoS One. 2022 Jan 5;17(1):e0261462. doi: 10.1371/journal.pone.0261462 (PMC8730409; doi:10.1371/journal.pone.0261462)
Supplement: S3 Table — Specific study arms are given in brackets. (DOCX) [file pone.0261462.s005.docx]

| **Fig 3C** | | | | | | | | | | | | | |
| --- | --- | --- | --- | --- | --- | --- | --- | --- | --- | --- | --- | --- | --- |
| ***low risk*** | | | | | | | ***high/unclear risk*** | | | | | | |
| Assmus 2013 (low dose) | | | | | | | Ang 2008 (ICI) | | | | | | |
| Assmus 2013 (high dose) | | | | | | | Ang 2008 (IMI) | | | | | | |
| Bartolucci 2017 | | | | | | | Duckers 2011 | | | | | | |
| Bartunek 2013 | | | | | | | Frljak 2018 | | | | | | |
| Bartunek 2017 (low dose) | | | | | | | Henry 2014 (ischemic) | | | | | | |
| Bartunek 2017 (mid dose) | | | | | | | Henry 2014 (non-ischemic) | | | | | | |
| Bartunek 2017 (high dose) | | | | | | | Henry 2017 | | | | | | |
| Brickwedel 2013 | | | | | | | Maureira 2012 | | | | | | |
| Makkar 2020 | | | | | | | Patel 2015 (ischemic) | | | | | | |
| Martino 2015 | | | | | | | Patel 2015 (non-ischemic) | | | | | | |
| Mathiasen 2015 | | | | | | | Perin 2012a | | | | | | |
| Menasche 2008 | | | | | | | Perin 2012b | | | | | | |
| Nasseri 2014 | | | | | | | Perin 2015 (25 mio) | | | | | | |
| Noiseux 2016 | | | | | | | Perin 2015 (75 mio) | | | | | | |
| Patel 2005 | | | | | | | Perin 2015 (150 mio) | | | | | | |
| Patel 2016 | | | | | | | Pokushalov 2010 | | | | | | |
| Perin 2011 | | | | | | | Sant'Anna 2014 | | | | | | |
| Santoso 2014 | | | | | | | Seth 2006 | | | | | | |
| Steinhoff 2017 | | | | | | | Trifunovic 2015 | | | | | | |
|  | | | | | | | Vrtovec 2011 | | | | | | |
|  | | | | | | | Wang 2015 | | | | | | |
|  | | | | | | | Xiao 2017 (BMMNC) | | | | | | |
|  | | | | | | | Xiao 2017 (BMMSC) | | | | | | |
|  | | | | | | | Zhao 2008 | | | | | | |
|  | | | | | | | Zhao 2015 | | | | | | |
|  | | | | | | |  | | | | | | |
| **Fig 3D** | | | | | | | | | | | | | |
| ***low risk*** | | | | | | | ***high/unclear risk*** | | | | | | |
| Bartolucci 2017 | | | | | | | Choudhury 2017 (ICI) | | | | | | |
| Bartunek 2017 (low dose) | | | | | | | Choudhury 2017 (TESI) | | | | | | |
| Bartunek 2017 (mid dose) | | | | | | | Henry 2014 (ischemic) | | | | | | |
| Bartunek 2017 (high dose) | | | | | | | Henry 2014 (non-ischemic) | | | | | | |
| Brickwedel 2013 | | | | | | | Hu 2011 | | | | | | |
| Heldman 2014 (BMMNC) | | | | | | | Patel 2015 (ischemic) | | | | | | |
| Heldman 2014 (BMMSC) | | | | | | | Patel 2015 (nonischemic) | | | | | | |
| Martino 2015 | | | | | | | Patila 2014 | | | | | | |
| Patel 2016 | | | | | | | Perin 2015 (25 mio) | | | | | | |
| Qi 2018 | | | | | | | Perin 2015 (75 mio) | | | | | | |
|  | | | | | | | Perin 2015 (150 mio) | | | | | | |
|  | | | | | | | Pokushalov 2010 | | | | | | |
|  | | | | | | | Sant'Anna 2014 | | | | | | |
|  | | | | | | | Trifunovic 2015 | | | | | | |
|  | | | | | | | Vrtovec 2011 | | | | | | |
|  | | | | | | | Xiao 2017 (BMMNC) | | | | | | |
|  | | | | | | | Xiao 2017 (BMMSC) | | | | | | |
|  | | | | | | |  | | | | | | |
| **Fig 3E** | | | | | | | | | | | | | |
| ***low risk*** | | | | | | | ***high/unclear risk*** | | | | | | |
| Ang 2008 (ICI) | | | | | | | Bartunek 2013 | | | | | | |
| Ang 2008 (IMI) | | | | | | | Duckers 2011 | | | | | | |
| Assmus 2013 (low dose) | | | | | | | Frljak 2018 | | | | | | |
| Assmus 2013 (high dose) | | | | | | | Henry 2014 (ischemic) | | | | | | |
| Bartolucci 2017 | | | | | | | Henry 2014 (non-ischemic) | | | | | | |
| Bartunek 2017 (low dose) | | | | | | | Maureira 2012 | | | | | | |
| Bartunek 2017 (mid dose) | | | | | | | Patel 2005 | | | | | | |
| Bartunek 2017 (high dose) | | | | | | | Patel 2015 (ischemic) | | | | | | |
| Brickwedel 2013 | | | | | | | Patel 2015 (non-ischemic) | | | | | | |
| Henry 2017 | | | | | | | Pokushalov 2010 | | | | | | |
| Makkar 2020 | | | | | | | Sant'Anna 2014 | | | | | | |
| Martino 2015 | | | | | | | Seth 2006 | | | | | | |
| Mathiasen 2015 | | | | | | | Trifunovic 2015 | | | | | | |
| Menasche 2008 | | | | | | | Vrtovec 2011 | | | | | | |
| Nasseri 2014 | | | | | | | Xiao 2017 (BMMNC) | | | | | | |
| Noiseux 2016 | | | | | | | Xiao 2017 (BMMSC) | | | | | | |
| Patel 2016 | | | | | | | Zhao 2008 | | | | | | |
| Perin 2011 | | | | | | | Zhao 2015 | | | | | | |
| Perin 2012a | | | | | | |  | | | | | | |
| Perin 2012b | | | | | | |  | | | | | | |
| Perin 2015 (25 mio) | | | | | | |  | | | | | | |
| Perin 2015 (75 mio) | | | | | | |  | | | | | | |
| Perin 2015 (150 mio) | | | | | | |  | | | | | | |
| Santoso 2014 | | | | | | |  | | | | | | |
| Steinhoff 2017 | | | | | | |  | | | | | | |
| Wang 2015 | | | | | | |  | | | | | | |
|  | | | | | | |  | | | | | | |
| **Fig 3F** | | | | | | | | | | | | | |
| ***low risk*** | | | | | | | ***high/unclear risk*** | | | | | | |
| Bartolucci 2017 | | | | | | | Henry 2014 (ischemic) | | | | | | |
| Bartunek 2017 (low dose) | | | | | | | Henry 2014 (non-ischemic) | | | | | | |
| Bartunek 2017 (mid dose) | | | | | | | Patel 2015 (ischemic) | | | | | | |
| Bartunek 2017 (high dose) | | | | | | | Patel 2015 (non-ischemic) | | | | | | |
| Brickwedel 2013 | | | | | | | Pokushalov 2010 | | | | | | |
| Choudhury 2017 (ICI) | | | | | | | Sant'Anna 2014 | | | | | | |
| Choudhury 2017 (TESI) | | | | | | | Trifunovic 2015 | | | | | | |
| Heldman 2014 (BMMNC) | | | | | | | Vrtovec 2011 | | | | | | |
| Heldman 2014 (BMMSC) | | | | | | | Xiao 2017 (BMMNC) | | | | | | |
| Hu 2011 | | | | | | | Xiao 2017 (BMMSC) | | | | | | |
| Martino 2015 | | | | | | |  | | | | | | |
| Patel 2016 | | | | | | |  | | | | | | |
| Patila 2014 | | | | | | |  | | | | | | |
| Perin 2015 (25 mio) | | | | | | |  | | | | | | |
| Perin 2015 (75 mio) | | | | | | |  | | | | | | |
| Perin 2015 (150 mio) | | | | | | |  | | | | | | |
| Qi 2018 | | | | | | |  | | | | | | |
|  | | | | | | |  | | | | | | |
| **Fig 3G** | | | | | | | | | | | | | |
| ***low risk*** | | | | | | | ***high/unclear risk*** | | | | | | |
| Ang 2008 (ICI) | | | | | | | Assmus 2013 (low dose) | | | | | | |
| Ang 2008 (IMI) | | | | | | | Assmus 2013 (high dose) | | | | | | |
| Bartolucci 2017 | | | | | | | Maureira 2012 | | | | | | |
| Bartunek 2013 | | | | | | | Patel 2015 (ischemic) | | | | | | |
| Bartunek 2017 (low dose) | | | | | | | Patel 2015 (non-ischemic) | | | | | | |
| Bartunek 2017 (mid dose) | | | | | | | Seth 2006 | | | | | | |
| Bartunek 2017 (high dose) | | | | | | | Trifunovic 2015 | | | | | | |
| Brickwedel 2013 | | | | | | | Zhao 2015 | | | | | | |
| Duckers 2011 | | | | | | |  | | | | | | |
| Frljak 2018 | | | | | | |  | | | | | | |
| Henry 2014 (ischemic) | | | | | | |  | | | | | | |
| Henry 2014 (non-ischemic) | | | | | | |  | | | | | | |
| Henry 2017 | | | | | | |  | | | | | | |
| Makkar 2020 | | | | | | |  | | | | | | |
| Martino 2015 | | | | | | |  | | | | | | |
| Mathiasen 2015 | | | | | | |  | | | | | | |
| Menasche 2008 | | | | | | |  | | | | | | |
| Nasseri 2014 | | | | | | |  | | | | | | |
| Noiseux 2016 | | | | | | |  | | | | | | |
| Patel 2005 | | | | | | |  | | | | | | |
| Patel 2016 | | | | | | |  | | | | | | |
| Perin 2011 | | | | | | |  | | | | | | |
| Perin 2012a | | | | | | |  | | | | | | |
| Perin 2012b | | | | | | |  | | | | | | |
| Perin 2015 (25 mio) | | | | | | |  | | | | | | |
| Perin 2015 (75 mio) | | | | | | |  | | | | | | |
| Perin 2015 (150 mio) | | | | | | |  | | | | | | |
| Pokushalov 2010 | | | | | | |  | | | | | | |
| Sant'Anna 2014 | | | | | | |  | | | | | | |
| Santoso 2014 | | | | | | |  | | | | | | |
| Steinhoff 2017 | | | | | | |  | | | | | | |
| Vrtovec 2011 | | | | | | |  | | | | | | |
| Wang 2015 | | | | | | |  | | | | | | |
| Xiao 2017 (BMMNC) | | | | | | |  | | | | | | |
| Xiao 2017 (BMMSC) | | | | | | |  | | | | | | |
| Zhao 2008 | | | | | | |  | | | | | | |
|  | | | | | | |  | | | | | | |
| **Fig 3H** | | | | | | | | | | | | | |
| ***low risk*** | | | | | | | ***high/unclear risk*** | | | | | | |
| Bartolucci 2017 | | | | | | | Patel 2015 (ischemic) | | | | | | |
| Bartunek 2017 (low dose) | | | | | | | Patel 2015 (non-ischemic) | | | | | | |
| Bartunek 2017 (mid dose) | | | | | | | Trifunovic 2015 | | | | | | |
| Bartunek 2017 (high dose) | | | | | | |  | | | | | | |
| Brickwedel 2013 | | | | | | |  | | | | | | |
| Choudhury 2017 (ICI) | | | | | | |  | | | | | | |
| Choudhury 2017 (TESI) | | | | | | |  | | | | | | |
| Heldman 2014 (BMMNC) | | | | | | |  | | | | | | |
| Heldman 2014 (BMMSC) | | | | | | |  | | | | | | |
| Henry 2014 (ischemic) | | | | | | |  | | | | | | |
| Henry 2014 (non-ischemic) | | | | | | |  | | | | | | |
| Hu 2011 | | | | | | |  | | | | | | |
| Martino 2015 | | | | | | |  | | | | | | |
| Patel 2016 | | | | | | |  | | | | | | |
| Patila 2014 | | | | | | |  | | | | | | |
| Perin 2015 (25 mio) | | | | | | |  | | | | | | |
| Perin 2015 (75 mio) | | | | | | |  | | | | | | |
| Perin 2015 (150 mio) | | | | | | |  | | | | | | |
| Pokushalov 2010 | | | | | | |  | | | | | | |
| Qi 2018 | | | | | | |  | | | | | | |
| Sant'Anna 2014 | | | | | | |  | | | | | | |
| Vrtovec 2011 | | | | | | |  | | | | | | |
| Xiao 2017 (BMMNC) | | | | | | |  | | | | | | |
| Xiao 2017 (BMMSC) | | | | | | |  | | | | | | |
|  | | | | | | |  | | | | | | |
| **Fig 4A** | | | | | | | | | | | | | |
| ***FUP < 6 months*** | | | | ***FUP > 6 to ≤ 12 months*** | | | | | | ***FUP > 12 months*** | | | |
| Ang 2008 | | | | Bartolucci 2017 | | | | | | Bartunek 2013 | | | |
| Assumus 2013 | | | | Bartunek 2017 | | | | | | Brickwedel 2013 | | | |
| Brickwedel 2013 | | | | Brickwedel 2013 | | | | | | Nasseri 2014 | | | |
| Duckers 2011 | | | | Choudhury 2017 (ICI) | | | | | | Patila 2014 | | | |
| Frljak 2018 | | | | Choudhury 2017 (TESI) | | | | | | Perin 2014 | | | |
| Henry 2014 (ischemic) | | | | Dib 2009 | | | | | | Perin 2015 (25 mio) | | | |
| Hu 2011 | | | | Heldman 2014 (BMMNC) | | | | | | Perin 2015 (75 mio) | | | |
| Martino 2015 | | | | Heldman 2014 (BMMSC) | | | | | | Perin 2015 (150 mio) | | | |
| Mathiasen 2015 | | | | Henry 2014 (non-ischemic) | | | | | | Pokushalov 2010 | | | |
| Maureira 2012 | | | | Henry 2017 | | | | | | Santoso 2014 | | | |
| Menasche 2008 | | | | Hu 2011 | | | | | | Seth 2006 | | | |
| Nasseri 2014 | | | | Makkar 2020 | | | | | | Steinhoff 2017 | | | |
| Noiseux 2016 | | | | Martino 2015 | | | | | | Trifunovic 2015 | | | |
| Patel 2005 | | | | Patel 2015 (ischemic) | | | | | | Vrtovec 2011 | | | |
| Perin 2011 | | | | Patel 2015 (non-ischemic) | | | | | |  | | | |
| Perin 2012a | | | | Patel 2016 | | | | | |  | | | |
| Perin 2012b | | | | Patila 2014 | | | | | |  | | | |
| Pokushalov 2010 | | | | Perin 2011 | | | | | |  | | | |
| Povsic 2011 | | | | Pokushalov 2010 | | | | | |  | | | |
| Sant'Anna 2014 | | | | Qi 2018 | | | | | |  | | | |
| Santoso 2014 | | | | Sant'Anna 2014 | | | | | |  | | | |
| Seth 2006 | | | | Steinhoff 2017 | | | | | |  | | | |
| Vrtovec 2011 | | | | Vrtovec 2011 | | | | | |  | | | |
| Wang 2015 | | | | Xiao 2017 (BMMNC) | | | | | |  | | | |
| Xiao 2017 (BMMNC) | | | | Xiao 2017 (BMMSC) | | | | | |  | | | |
| Xiao 2017 (BMMSC) | | | |  | | | | | |  | | | |
| Zhao 2008 | | | |  | | | | | |  | | | |
| Zhao 2015 | | | |  | | | | | |  | | | |
|  | | | | | | |  | | | | | | |
| **Fig 4B** | | | | | | | | | | | | | |
| ***FUP < 6 months*** | | | | ***FUP > 6 to ≤ 12 months*** | | | | | | ***FUP > 12 months*** | | | |
| Ang 2008 (ICI) | | | | Bartolucci 2017 | | | | | | Menasche 2008 | | | |
| Ang 2008 (IMI) | | | | Heldman 2014 (BMMNC) | | | | | | Perin 2014 | | | |
| Assumus 2013 | | | | Heldman 2014 (BMMSC) | | | | | | Perin 2015 (25 mio) | | | |
| Duckers 2011 | | | | Henry 2014 (ischemic) | | | | | | Perin 2015 (75 mio) | | | |
| Makkar 2020 | | | | Henry 2014 (non-ischemic) | | | | | | Perin 2015 (150 mio) | | | |
| Maureira 2012 | | | | Henry 2017 | | | | | |  | | | |
| Menasche 2008 | | | | Hu 2011 | | | | | |  | | | |
| Noiseux 2016 | | | | Makkar 2020 | | | | | |  | | | |
| Patila 2014 | | | | Martino 2015 | | | | | |  | | | |
| Perin 2012b | | | | Menasche 2008 | | | | | |  | | | |
| Posvic 2011 (low dose) | | | | Patel 2015 (ischemic) | | | | | |  | | | |
| Povsic 2011 (high dose) | | | | Patel 2015 (non-ischemic) | | | | | |  | | | |
| Steinhoff 2017 | | | | Patel 2016 | | | | | |  | | | |
|  | | | | Patila 2014 | | | | | |  | | | |
|  | | | | Xiao 2017 (BMMNC) | | | | | |  | | | |
|  | | | | Xiao 2017 (BMMSC) | | | | | |  | | | |
|  | | | | | | |  | | | | | | |
| **Fig 4C** | | | | | | | | | | | | | |
| ***FUP < 6 months*** | | | | ***FUP > 6 to ≤ 12 months*** | | | | | | ***FUP > 12 months*** | | | |
| Ang 2008 | | | | Bartunek 2017 | | | | | | Menasche 2008 | | | |
| Dib 2009 | | | | Heldman 2014 (BMMNC) | | | | | |  | | | |
| Duckers 2011 | | | | Heldman 2014 (BMMSC) | | | | | |  | | | |
| Makkar 2020 | | | | Henry 2017 | | | | | |  | | | |
| Menasche 2008 | | | | Makkar 2020 | | | | | |  | | | |
| Nasseri 2014 | | | | Menasche 2008 | | | | | |  | | | |
| Noiseux 2016 | | | | Patel 2015 (ischemic) | | | | | |  | | | |
| Perin 2012b | | | | Patel 2015 (non-ischemic) | | | | | |  | | | |
| Posvic 2011 (low dose) | | | | Patel 2016 | | | | | |  | | | |
| Povsic 2011 (high dose) | | | | Perin 2011 | | | | | |  | | | |
| Steinhoff 2017 | | | |  | | | | | |  | | | |
|  | | | | | | |  | | | | | | |
| **Fig 4D** | | | | | | | | | | | | | |
| ***FUP < 6 months*** | | | | | | | ***FUP > 6 to ≤ 12 months*** | | | | | | |
| Ang 2008 (ICI) | | | | | | | Bartolucci 2017 | | | | | | |
| Ang 2008 (IMI) | | | | | | | Bartunek 2017 (low dose) | | | | | | |
| Assmus 2013 (low dose) | | | | | | | Bartunek 2017 (mid dose) | | | | | | |
| Assmus 2013 (high dose) | | | | | | | Bartunek 2017 (high dose) | | | | | | |
| Bartolucci 2017 | | | | | | | Brickwedel 2013 | | | | | | |
| Bartunek 2013 | | | | | | | Choudhury 2017 (ICI) | | | | | | |
| Bartunek 2017 (low dose) | | | | | | | Choudhury 2017 (TESI) | | | | | | |
| Bartunek 2017 (mid dose) | | | | | | | Heldman 2014 (BMMNC) | | | | | | |
| Bartunek 2017 (high dose) | | | | | | | Heldman 2014 (BMMSC) | | | | | | |
| Brickwedel 2013 | | | | | | | Henry 2014 (ischemic) | | | | | | |
| Duckers 2011 | | | | | | | Henry 2014 (non-ischemic) | | | | | | |
| Frljak 2018 | | | | | | | Hu 2011 | | | | | | |
| Henry 2014 (ischemic) | | | | | | | Martino 2015 | | | | | | |
| Henry 2014 (non-ischemic) | | | | | | | Patel 2015 (ischemic) | | | | | | |
| Henry 2017 | | | | | | | Patel 2015 (non-ischemic) | | | | | | |
| Makkar 2020 | | | | | | | Patel 2016 | | | | | | |
| Martino 2015 | | | | | | | Patila 2014 | | | | | | |
| Mathiasen 2015 | | | | | | | Perin 2015 (25 mio) | | | | | | |
| Maureira 2012 | | | | | | | Perin 2015 (75 mio) | | | | | | |
| Menasche 2008 | | | | | | | Perin 2015 (150 mio) | | | | | | |
| Nasseri 2014 | | | | | | | Pokushalov 2010 | | | | | | |
| Noiseux 2016 | | | | | | | Qi 2018 | | | | | | |
| Patel 2005 | | | | | | | Sant'Anna 2014 | | | | | | |
| Patel 2015 (ischemic) | | | | | | | Trifunovic 2015 | | | | | | |
| Patel 2015 (non-ischemic) | | | | | | | Vrtovec 2011 | | | | | | |
| Patel 2016 | | | | | | | Xiao 2017 (BMMNC) | | | | | | |
| Perin 2011 | | | | | | | Xiao 2017 (BMMSC) | | | | | | |
| Perin 2012a | | | | | | |  | | | | | | |
| Perin 2012b | | | | | | |  | | | | | | |
| Perin 2015 (25 mio) | | | | | | |  | | | | | | |
| Perin 2015 (75 mio) | | | | | | |  | | | | | | |
| Perin 2015 (150 mio) | | | | | | |  | | | | | | |
| Pokushalov 2010 | | | | | | |  | | | | | | |
| Sant'Anna 2014 | | | | | | |  | | | | | | |
| Santoso 2014 | | | | | | |  | | | | | | |
| Seth 2006 | | | | | | |  | | | | | | |
| Steinhoff 2017 | | | | | | |  | | | | | | |
| Trifunovic 2015 | | | | | | |  | | | | | | |
| Vrtovec 2011 | | | | | | |  | | | | | | |
| Wang 2015 | | | | | | |  | | | | | | |
| Xiao 2017 (BMMNC) | | | | | | |  | | | | | | |
| Xiao 2017 (BMMSC) | | | | | | |  | | | | | | |
| Zhao 2008 | | | | | | |  | | | | | | |
| Zhao 2015 | | | | | | |  | | | | | | |
|  | | | | | | |  | | | | | | |
| **Fig 4E** | | | | | | | | | | | | | |
| ***FUP < 6 months*** | | | | | | | ***FUP > 6 to ≤ 12 months*** | | | | | | |
| Ang 2008 (ICI) | | | | | | | Bartolucci 2017 | | | | | | |
| Ang 2008 (IMI) | | | | | | | Bartunek 2017 (low dose) | | | | | | |
| Assmus 2013 (low dose) | | | | | | | Bartunek 2017 (mid dose) | | | | | | |
| Assmus 2013 (high dose) | | | | | | | Bartunek 2017 (high dose) | | | | | | |
| Bartolucci 2017 | | | | | | | Brickwedel 2013 | | | | | | |
| Bartunek 2013 | | | | | | | Choudhury 2017 (ICI) | | | | | | |
| Bartunek 2017 (low dose) | | | | | | | Choudhury 2017 (TESI) | | | | | | |
| Bartunek 2017 (mid dose) | | | | | | | Dib 2009 | | | | | | |
| Bartunek 2017 (high dose) | | | | | | | Heldman 2014 (BMMNC) | | | | | | |
| Brickwedel 2013 | | | | | | | Heldman 2014 (BMMSC) | | | | | | |
| Frljak 2018 | | | | | | | Henry 2014 (ischemic) | | | | | | |
| Henry 2014 (ischemic) | | | | | | | Henry 2014 (non-ischemic) | | | | | | |
| Henry 2014 (non-ischemic) | | | | | | | Hu 2011 | | | | | | |
| Henry 2017 | | | | | | | Martino 2015 | | | | | | |
| Makkar 2020 | | | | | | | Patel 2016 | | | | | | |
| Martino 2015 | | | | | | | Perin 2015 (25 mio) | | | | | | |
| Mathiasen 2015 | | | | | | | Perin 2015 (75 mio) | | | | | | |
| Maureira 2012 | | | | | | | Perin 2015 (150 mio) | | | | | | |
| Nasseri 2014 | | | | | | | Pokushalov 2010 | | | | | | |
| Noiseux 2016 | | | | | | | Sant'Anna 2014 | | | | | | |
| Patel 2005 | | | | | | | Vrtovec 2011 | | | | | | |
| Patel 2016 | | | | | | |  | | | | | | |
| Perin 2011 | | | | | | |  | | | | | | |
| Perin 2012a | | | | | | |  | | | | | | |
| Perin 2012b | | | | | | |  | | | | | | |
| Perin 2015 (25 mio) | | | | | | |  | | | | | | |
| Perin 2015 (75 mio) | | | | | | |  | | | | | | |
| Perin 2015 (150 mio) | | | | | | |  | | | | | | |
| Pokushalov 2010 | | | | | | |  | | | | | | |
| Sant'Anna 2014 | | | | | | |  | | | | | | |
| Santoso 2014 | | | | | | |  | | | | | | |
| Steinhoff 2017 | | | | | | |  | | | | | | |
| Vrtovec 2011 | | | | | | |  | | | | | | |
|  | | | | | | |  | | | | | | |
| **Fig 4F** | | | | | | | | | | | | | |
| ***FUP < 6 months*** | | | | | | | ***FUP > 6 to ≤ 12 months*** | | | | | | |
| Assmus 2013 (low dose) | | | | | | | Bartolucci 2017 | | | | | | |
| Assmus 2013 (high dose) | | | | | | | Choudhury 2017 (ICI) | | | | | | |
| Bartolucci 2017 | | | | | | | Choudhury 2017 (TESI) | | | | | | |
| Choudhury 2017 (ICI) | | | | | | | Dib 2009 | | | | | | |
| Choudhury 2017 (TESI) | | | | | | | Perin 2014 | | | | | | |
| Dib 2009 | | | | | | | Pokushalov 2010 | | | | | | |
| Duckers 2011 | | | | | | | Sant'Anna 2014 | | | | | | |
| Mathiasen 2015 | | | | | | | Seth 2006 | | | | | | |
| Nasseri 2014 | | | | | | | Trifunovic 2015 | | | | | | |
| Patel 2005 | | | | | | | Xiao 2017 (BMMNC) | | | | | | |
| Perin 2011 | | | | | | | Xiao 2017 (BMMSC) | | | | | | |
| Perin 2012a | | | | | | |  | | | | | | |
| Perin 2014 | | | | | | |  | | | | | | |
| Pokushalov 2010 | | | | | | |  | | | | | | |
| Sant'Anna 2014 | | | | | | |  | | | | | | |
| Trifunovic 2015 | | | | | | |  | | | | | | |
| Xiao 2017 (BMMNC) | | | | | | |  | | | | | | |
| Xiao 2017 (BMMSC) | | | | | | |  | | | | | | |
| Zhao 2008 | | | | | | |  | | | | | | |
|  | | | | | | |  | | | | | | |
| **Fig 4G** | | | | | | | | | | | | | |
| ***FUP < 6 months*** | | | | | | | ***FUP > 6 to ≤ 12 months*** | | | | | | |
| Bartunek 2013 | | | | | | | Heldman 2014 (BMMNC) | | | | | | |
| Duckers 2011 | | | | | | | Heldman 2014 (BMMSC) | | | | | | |
| Frljak 2018 | | | | | | | Henry 2014 (ischemic) | | | | | | |
| Heldman 2014 (BMMNC) | | | | | | | Henry 2014 (non-ischemic) | | | | | | |
| Heldman 2014 (BMMSC) | | | | | | | Martino 2015 | | | | | | |
| Henry 2014 (ischemic) | | | | | | | Sant'Anna 2014 | | | | | | |
| Henry 2014 (non-ischemic) | | | | | | | Vrtovec 2011 | | | | | | |
| Hu 2011 | | | | | | |  | | | | | | |
| Makkar 2020 | | | | | | |  | | | | | | |
| Martino 2015 | | | | | | |  | | | | | | |
| Perin 2012b | | | | | | |  | | | | | | |
| Posvic 2011 (low dose) | | | | | | |  | | | | | | |
| Povsic 2011 (high dose) | | | | | | |  | | | | | | |
| Sant'Anna 2014 | | | | | | |  | | | | | | |
| Steinhoff 2017 | | | | | | |  | | | | | | |
|  | | | | | | |  | | | | | | |
| **Fig 4H** | | | | | | | | | | | | | |
| ***FUP < 6 months*** | | | | | | | ***FUP > 6 to ≤ 12 months*** | | | | | | |
| Frljak 2018 | | | | | | | Vrtovec 2011 | | | | | | |
| Makkar 2020 | | | | | | |  | | | | | | |
| Perin 2012b | | | | | | |  | | | | | | |
| Vrtovec 2011 | | | | | | |  | | | | | | |
|  | | | | | | |  | | | | | | |
| **Fig 4I** | | | | | | | | | | | | | |
| ***FUP < 6 months*** | | | | | | | ***FUP > 6 to ≤ 12 months*** | | | | | | |
| Bartolucci 2017 | | | | | | | Bartolucci 2017 | | | | | | |
| Choudhury 2017 (ICI) | | | | | | | Choudhury 2017 (ICI) | | | | | | |
| Choudhury 2017 (TESI) | | | | | | | Choudhury 2017 (TESI) | | | | | | |
| Dib 2009 | | | | | | | Dib 2009 | | | | | | |
| Duckers 2011 | | | | | | | Martino 2015 | | | | | | |
| Makkar 2020 | | | | | | | Pokushalov 2010 | | | | | | |
| Martino 2015 | | | | | | | Sant'Anna 2014 | | | | | | |
| Perin 2011 | | | | | | |  | | | | | | |
| Pokushalov 2010 | | | | | | |  | | | | | | |
| Sant'Anna 2014 | | | | | | |  | | | | | | |
|  | | | | | | |  | | | | | | |
| **Fig 5A** | | | | | | | | | | | | | |
| ***primary cells*** | | | | | | | ***cultured cells*** | | | | | | |
| Ang 2008 | | | | | | | Brickwedel 2013 | | | | | | |
| Assumus 2013 | | | | | | | Duckers 2011 | | | | | | |
| Frljak 2018 | | | | | | | Henry 2014 (ischemic) | | | | | | |
| Hu 2011 | | | | | | | Mathiasen 2015 | | | | | | |
| Martino 2015 | | | | | | | Menasche 2008 | | | | | | |
| Maureira 2012 | | | | | | | Povsic 2011 | | | | | | |
| Nasseri 2014 | | | | | | | Xiao 2017 (BMMSC) | | | | | | |
| Noiseux 2016 | | | | | | | Zhao 2015 | | | | | | |
| Patel 2005 | | | | | | |  | | | | | | |
| Perin 2011 | | | | | | |  | | | | | | |
| Perin 2012a | | | | | | |  | | | | | | |
| Perin 2012b | | | | | | |  | | | | | | |
| Pokushalov 2010 | | | | | | |  | | | | | | |
| Sant'Anna 2014 | | | | | | |  | | | | | | |
| Santoso 2014 | | | | | | |  | | | | | | |
| Seth 2006 | | | | | | |  | | | | | | |
| Vrtovec 2011 | | | | | | |  | | | | | | |
| Wang 2015 | | | | | | |  | | | | | | |
| Xiao 2017 (BMMNC) | | | | | | |  | | | | | | |
| Zhao 2008 | | | | | | |  | | | | | | |
|  | | | | | | |  | | | | | | |
| **Fig 5B** | | | | | | | | | | | | | |
| ***primary cells*** | | | | | | | ***cultured cells*** | | | | | | |
| Choudhury 2017 (ICI) | | | | | | | Bartolucci 2017 | | | | | | |
| Choudhury 2017 (TESI) | | | | | | | Bartunek 2017 | | | | | | |
| Heldman 2014 (BMMNC) | | | | | | | Brickwedel 2013 | | | | | | |
| Henry 2017 | | | | | | | Dib 2009 | | | | | | |
| Hu 2011 | | | | | | | Heldman 2014 (BMMSC | | | | | | |
| Makkar 2020 | | | | | | | Henry 2014 (non-ischemic) | | | | | | |
| Martino 2015 | | | | | | | Makkar 2020 | | | | | | |
| Patel 2015 (ischemic) | | | | | | | Patel 2016 | | | | | | |
| Patel 2015 (non-ischemic) | | | | | | | Xiao 2017 (BMMSC) | | | | | | |
| Patila 2014 | | | | | | |  | | | | | | |
| Perin 2011 | | | | | | |  | | | | | | |
| Pokushalov 2010 | | | | | | |  | | | | | | |
| Qi 2018 | | | | | | |  | | | | | | |
| Sant'Anna 2014 | | | | | | |  | | | | | | |
| Steinhoff 2017 | | | | | | |  | | | | | | |
| Vrtovec 2011 | | | | | | |  | | | | | | |
| Xiao 2017 (BMMNC) | | | | | | |  | | | | | | |
|  | | | | | | |  | | | | | | |
| **Fig 5C** | | | | | | | | | | | | | |
| ***primary cells*** | | | | | | | ***cultured cells*** | | | | | | |
| Nasseri 2014 | | | | | | | Bartunek 2013 | | | | | | |
| Patila 2014 | | | | | | | Brickwedel 2013 | | | | | | |
| Perin 2014 | | | | | | | Perin 2015 (25 mio) | | | | | | |
| Pokushalov 2010 | | | | | | | Perin 2015 (75 mio) | | | | | | |
| Santoso 2014 | | | | | | | Perin 2015 (150 mio) | | | | | | |
| Seth 2006 | | | | | | |  | | | | | | |
| Steinhoff 2017 | | | | | | |  | | | | | | |
| Trifunovic 2015 | | | | | | |  | | | | | | |
| Vrtovec 2011 | | | | | | |  | | | | | | |
|  | | | | | | |  | | | | | | |
| **Fig 5D** | | | | | | | | | | | | | |
| ***primary cells*** | | | | | | | ***cultured cells*** | | | | | | |
| Ang 2008 (ICI) | | | | | | | Bartolucci 2017 | | | | | | |
| Ang 2008 (IMI) | | | | | | | Bartunek 2013 | | | | | | |
| Assmus 2013 (low dose) | | | | | | | Bartunek 2017 (low dose) | | | | | | |
| Assmus 2013 (high dose) | | | | | | | Bartunek 2017 (mid dose) | | | | | | |
| Frljak 2018 | | | | | | | Bartunek 2017 (high dose) | | | | | | |
| Henry 2017 | | | | | | | Brickwedel 2013 | | | | | | |
| Martino 2015 | | | | | | | Duckers 2011 | | | | | | |
| Maureira 2012 | | | | | | | Henry 2014 (ischemic) | | | | | | |
| Nasseri 2014 | | | | | | | Henry 2014 (non-ischemic) | | | | | | |
| Noiseux 2016 | | | | | | | Makkar 2020 | | | | | | |
| Patel 2005 | | | | | | | Mathiasen 2015 | | | | | | |
| Patel 2015 (ischemic) | | | | | | | Menasche 2008 | | | | | | |
| Patel 2015 (non-ischemic) | | | | | | | Patel 2016 | | | | | | |
| Perin 2011 | | | | | | | Perin 2015 (25 mio) | | | | | | |
| Perin 2012a | | | | | | | Perin 2015 (75 mio) | | | | | | |
| Perin 2012b | | | | | | | Perin 2015 (150 mio) | | | | | | |
| Pokushalov 2010 | | | | | | | Xiao 2017 (BMMSC) | | | | | | |
| Sant'Anna 2014 | | | | | | | Zhao 2015 | | | | | | |
| Santoso 2014 | | | | | | |  | | | | | | |
| Seth 2006 | | | | | | |  | | | | | | |
| Steinhoff 2017 | | | | | | |  | | | | | | |
| Trifunovic 2015 | | | | | | |  | | | | | | |
| Vrtovec 2011 | | | | | | |  | | | | | | |
| Wang 2015 | | | | | | |  | | | | | | |
| Xiao 2017 (BMMNC) | | | | | | |  | | | | | | |
| Zhao 2008 | | | | | | |  | | | | | | |
|  | | | | | | |  | | | | | | |
| **Fig 5E** | | | | | | | | | | | | | |
| ***primary cells*** | | | | | | | ***cultured cells*** | | | | | | |
| Choudhury 2017 (ICI) | | | | | | | Bartolucci 2017 | | | | | | |
| Choudhury 2017 (TESI) | | | | | | | Bartunek 2017 (low dose) | | | | | | |
| Heldman 2014 (BMMNC) | | | | | | | Bartunek 2017 (mid dose) | | | | | | |
| Hu 2011 | | | | | | | Bartunek 2017 (high dose) | | | | | | |
| Martino 2015 | | | | | | | Brickwedel 2013 | | | | | | |
| Patel 2015 (ischemic) | | | | | | | Heldman 2014 (BMMSC) | | | | | | |
| Patel 2015 (non-ischemic) | | | | | | | Henry 2014 (ischemic) | | | | | | |
| Patila 2014 | | | | | | | Henry 2014 (non-ischemic) | | | | | | |
| Pokushalov 2010 | | | | | | | Patel 2016 | | | | | | |
| Qi 2018 | | | | | | | Perin 2015 (25 mio) | | | | | | |
| Sant'Anna 2014 | | | | | | | Perin 2015 (75 mio) | | | | | | |
| Trifunovic 2015 | | | | | | | Perin 2015 (150 mio) | | | | | | |
| Vrtovec 2011 | | | | | | | Xiao 2017 (BMMSC) | | | | | | |
| Xiao 2017 (BMMNC) | | | | | | |  | | | | | | |
|  | | | | | | |  | | | | | | |
| **Fig 5F** | | | | | | | | | | | | | |
| ***primary cells*** | | | | | | | ***cultured cells*** | | | | | | |
| Ang 2008 (ICI) | | | | | | | Bartolucci 2017 | | | | | | |
| Ang 2008 (IMI) | | | | | | | Bartunek 2013 | | | | | | |
| Assmus 2013 (low dose) | | | | | | | Bartunek 2017 (low dose) | | | | | | |
| Assmus 2013 (high dose) | | | | | | | Bartunek 2017 (mid dose) | | | | | | |
| Frljak 2018 | | | | | | | Bartunek 2017 (high dose) | | | | | | |
| Henry 2017 | | | | | | | Brickwedel 2013 | | | | | | |
| Martino 2015 | | | | | | | Henry 2014 (ischemic) | | | | | | |
| Maureira 2012 | | | | | | | Henry 2014 (non-ischemic) | | | | | | |
| Nasseri 2014 | | | | | | | Makkar 2020 | | | | | | |
| Noiseux 2016 | | | | | | | Mathiasen 2015 | | | | | | |
| Patel 2005 | | | | | | | Patel 2016 | | | | | | |
| Perin 2011 | | | | | | | Perin 2015 (25 mio) | | | | | | |
| Perin 2012a | | | | | | | Perin 2015 (75 mio) | | | | | | |
| Perin 2012b | | | | | | | Perin 2015 (150 mio) | | | | | | |
| Pokushalov 2010 | | | | | | |  | | | | | | |
| Sant'Anna 2014 | | | | | | |  | | | | | | |
| Santoso 2014 | | | | | | |  | | | | | | |
| Steinhoff 2017 | | | | | | |  | | | | | | |
| Vrtovec 2011 | | | | | | |  | | | | | | |
|  | | | | | | |  | | | | | | |
| **Fig 5G** | | | | | | | | | | | | | |
| ***primary cells*** | | | | | | | ***cultured cells*** | | | | | | |
| Choudhury 2017 (ICI) | | | | | | | Bartolucci 2017 | | | | | | |
| Choudhury 2017 (TESI) | | | | | | | Bartunek 2017 (low dose) | | | | | | |
| Heldman 2014 (BMMNC) | | | | | | | Bartunek 2017 (mid dose) | | | | | | |
| Hu 2011 | | | | | | | Bartunek 2017 (high dose) | | | | | | |
| Martino 2015 | | | | | | | Brickwedel 2013 | | | | | | |
| Pokushalov 2010 | | | | | | | Dib 2009 | | | | | | |
| Sant'Anna 2014 | | | | | | | Heldman 2014 BMMSC | | | | | | |
| Vrtovec 2011 | | | | | | | Henry 2014 ischemic arm | | | | | | |
|  | | | | | | | Henry 2014 non-ischemic arm | | | | | | |
|  | | | | | | | Patel 2016 | | | | | | |
|  | | | | | | | Perin 2015 (25 mio) | | | | | | |
|  | | | | | | | Perin 2015 (75 mio) | | | | | | |
|  | | | | | | | Perin 2015 (150 mio) | | | | | | |
|  | | | | | | |  | | | | | | |
| **Fig 5H** | | | | | | | | | | | | | |
| ***primary cells*** | | | | | | | ***cultured cells*** | | | | | | |
| Assmus 2013 (low dose) | | | | | | | Bartolucci 2017 | | | | | | |
| Assmus 2013 (high dose) | | | | | | | Dib 2009 | | | | | | |
| Choudhury 2017 (ICI) | | | | | | | Duckers 2011 | | | | | | |
| Choudhury 2017 (TESI) | | | | | | | Mathiasen 2015 | | | | | | |
| Nasseri 2014 | | | | | | | Xiao 2017 (BMMSC) | | | | | | |
| Patel 2005 | | | | | | |  | | | | | | |
| Perin 2011 | | | | | | |  | | | | | | |
| Perin 2012a | | | | | | |  | | | | | | |
| Perin 2014 | | | | | | |  | | | | | | |
| Pokushalov 2010 | | | | | | |  | | | | | | |
| Sant'Anna 2014 | | | | | | |  | | | | | | |
| Trifunovic 2015 | | | | | | |  | | | | | | |
| Xiao 2017 (BMMNC) | | | | | | |  | | | | | | |
| Zhao 2008 | | | | | | |  | | | | | | |
|  | | | | | | |  | | | | | | |
| **Fig 5I** | | | | | | | | | | | | | |
| ***primary cells*** | | | | | | | ***cultured cells*** | | | | | | |
| Choudhury 2017 (ICI) | | | | | | | Bartolucci 2017 | | | | | | |
| Choudhury 2017 (TESI) | | | | | | | Dib 2009 | | | | | | |
| Perin 2014 | | | | | | | Xiao 2017 (BMMSC) | | | | | | |
| Pokushalov 2010 | | | | | | |  | | | | | | |
| Sant'Anna 2014 | | | | | | |  | | | | | | |
| Seth 2006 | | | | | | |  | | | | | | |
| Trifunovic 2015 | | | | | | |  | | | | | | |
| Xiao 2017 (BMMNC) | | | | | | |  | | | | | | |
|  | | | | | | |  | | | | | | |
| **Fig 6A** | | | | | | | | | | | | | |
| ***muscle-derived*** | | ***BM-derived*** | | | | ***AT-derived*** | | ***perinatal*** | | | | ***cardiac-derived*** | |
| Brickwedel 2013 | | Ang 2008 | | | | – | | Zhao 2015 | | | | – | |
| Duckers 2011 | | Assumus 2013 | | | |  | |  | | | |  | |
| Menasche 2008 | | Frljak 2018 | | | |  | |  | | | |  | |
| Povsic 2011 | | Henry 2014 (ischemic) | | | |  | |  | | | |  | |
|  | | Hu 2011 | | | |  | |  | | | |  | |
|  | | Martino 2015 | | | |  | |  | | | |  | |
|  | | Mathiasen 2015 | | | |  | |  | | | |  | |
|  | | Maureira 2012 | | | |  | |  | | | |  | |
|  | | Nasseri 2014 | | | |  | |  | | | |  | |
|  | | Noiseux 2016 | | | |  | |  | | | |  | |
|  | | Patel 2005 | | | |  | |  | | | |  | |
|  | | Perin 2011 | | | |  | |  | | | |  | |
|  | | Perin 2012a | | | |  | |  | | | |  | |
|  | | Perin 2012b | | | |  | |  | | | |  | |
|  | | Pokushalov 2010 | | | |  | |  | | | |  | |
|  | | Sant'Anna 2014 | | | |  | |  | | | |  | |
|  | | Santoso 2014 | | | |  | |  | | | |  | |
|  | | Seth 2006 | | | |  | |  | | | |  | |
|  | | Vrtovec 2011 | | | |  | |  | | | |  | |
|  | | Wang 2015 | | | |  | |  | | | |  | |
|  | | Xiao 2017 (BMMNC) | | | |  | |  | | | |  | |
|  | | Xiao 2017 (BMMSC) | | | |  | |  | | | |  | |
|  | | Zhao 2008 | | | |  | |  | | | |  | |
|  | |  | | | |  | |  | | | |  | |
| **Fig 6B** | | | | | | | | | | | | | |
| ***muscle-derived*** | | ***BM-derived*** | | | | ***AT-derived*** | | ***perinatal*** | | | | ***cardiac-derived*** | |
| Brickwedel 2013 | | Bartunek 2017 | | | | Henry 2017 | | Bartolucci 2017 | | | | Makkar 2020 | |
| Dib 2009 | | Choudhury 2017 (ICI) | | | |  | |  | | | |  | |
|  | | Choudhury 2017 (TESI) | | | |  | |  | | | |  | |
|  | | Heldman 2014 (BMMNC) | | | |  | |  | | | |  | |
|  | | Heldman 2014 (BMMSC) | | | |  | |  | | | |  | |
|  | | Henry 2014 (non-ischemic) | | | |  | |  | | | |  | |
|  | | Hu 2011 | | | |  | |  | | | |  | |
|  | | Martino 2015 | | | |  | |  | | | |  | |
|  | | Patel 2015 (ischemic) | | | |  | |  | | | |  | |
|  | | Patel 2015 (non-ischemic) | | | |  | |  | | | |  | |
|  | | Patel 2016 | | | |  | |  | | | |  | |
|  | | Patila 2014 | | | |  | |  | | | |  | |
|  | | Perin 2011 | | | |  | |  | | | |  | |
|  | | Pokushalov 2010 | | | |  | |  | | | |  | |
|  | | Qi 2018 | | | |  | |  | | | |  | |
|  | | Sant'Anna 2014 | | | |  | |  | | | |  | |
|  | | Steinhoff 2017 | | | |  | |  | | | |  | |
|  | | Vrtovec 2011 | | | |  | |  | | | |  | |
|  | | Xiao 2017 (BMMNC) | | | |  | |  | | | |  | |
|  | |  | | | |  | |  | | | |  | |
| **Fig 6C** | | | | | | | | | | | | | |
| ***muscle-derived*** | | ***BM-derived*** | | | | ***AT-derived*** | | ***perinatal*** | | | | ***cardiac-derived*** | |
| Brickwedel 2013 | | Bartunek 2013 | | | | Perin 2014 | | – | | | | – | |
|  | | Nasseri 2014 | | | |  | |  | | | |  | |
|  | | Patila 2014 | | | |  | |  | | | |  | |
|  | | Perin 2015 (25 mio) | | | |  | |  | | | |  | |
|  | | Perin 2015 (75 mio) | | | |  | |  | | | |  | |
|  | | Perin 2015 (150 mio) | | | |  | |  | | | |  | |
|  | | Pokushalov 2010 | | | |  | |  | | | |  | |
|  | | Santoso 2014 | | | |  | |  | | | |  | |
|  | | Seth 2006 | | | |  | |  | | | |  | |
|  | | Steinhoff 2017 | | | |  | |  | | | |  | |
|  | | Trifunovic 2015 | | | |  | |  | | | |  | |
|  | | Vrtovec 2011 | | | |  | |  | | | |  | |
|  | |  | | | |  | |  | | | |  | |
| **Fig 6D** | | | | | | | | | | | | | |
| ***muscle-derived*** | | ***BM-derived*** | | | | ***AT-derived*** | | ***perinatal*** | | | | ***cardiac-derived*** | |
| Brickwedel 2013 | | Ang 2008 (ICI) | | | | Henry 2017 | | Bartolucci 2017 | | | | Makkar 2020 | |
| Duckers 2011 | | Ang 2008 (IMI) | | | |  | | Zhao 2015 | | | |  | |
| Menasche 2008 | | Assmus 2013 (low dose) | | | |  | |  | | | |  | |
|  | | Assmus 2013 (high dose) | | | |  | |  | | | |  | |
|  | | Bartunek 2013 | | | |  | |  | | | |  | |
|  | | Bartunek 2017 (low dose) | | | |  | |  | | | |  | |
|  | | Bartunek 2017 (mid dose) | | | |  | |  | | | |  | |
|  | | Bartunek 2017 (high dose) | | | |  | |  | | | |  | |
|  | | Frljak 2018 | | | |  | |  | | | |  | |
|  | | Henry 2014 (ischemic) | | | |  | |  | | | |  | |
|  | | Henry 2014 (non-ischemic) | | | |  | |  | | | |  | |
|  | | Martino 2015 | | | |  | |  | | | |  | |
|  | | Mathiasen 2015 | | | |  | |  | | | |  | |
|  | | Maureira 2012 | | | |  | |  | | | |  | |
|  | | Nasseri 2014 | | | |  | |  | | | |  | |
|  | | Noiseux 2016 | | | |  | |  | | | |  | |
|  | | Patel 2005 | | | |  | |  | | | |  | |
|  | | Patel 2015 (ischemic) | | | |  | |  | | | |  | |
|  | | Patel 2015 (non-ischemic) | | | |  | |  | | | |  | |
|  | | Patel 2016 | | | |  | |  | | | |  | |
|  | | Perin 2011 | | | |  | |  | | | |  | |
|  | | Perin 2012a | | | |  | |  | | | |  | |
|  | | Perin 2012b | | | |  | |  | | | |  | |
|  | | Perin 2015 (25 mio) | | | |  | |  | | | |  | |
|  | | Perin 2015 (75 mio) | | | |  | |  | | | |  | |
|  | | Perin 2015 (150 mio) | | | |  | |  | | | |  | |
|  | | Pokushalov 2010 | | | |  | |  | | | |  | |
|  | | Sant'Anna 2014 | | | |  | |  | | | |  | |
|  | | Santoso 2014 | | | |  | |  | | | |  | |
|  | | Seth 2006 | | | |  | |  | | | |  | |
|  | | Steinhoff 2017 | | | |  | |  | | | |  | |
|  | | Trifunovic 2015 | | | |  | |  | | | |  | |
|  | | Vrtovec 2011 | | | |  | |  | | | |  | |
|  | | Wang 2015 | | | |  | |  | | | |  | |
|  | | Xiao 2017 (BMMNC) | | | |  | |  | | | |  | |
|  | | Xiao 2017 (BMMSC) | | | |  | |  | | | |  | |
|  | | Zhao 2008 | | | |  | |  | | | |  | |
|  | |  | | | |  | |  | | | |  | |
| **Fig 6E** | | | | | | | | | | | | | |
| ***muscle-derived*** | | ***BM-derived*** | | | | ***AT-derived*** | | ***perinatal*** | | | | ***cardiac-derived*** | |
| Brickwedel 2013 | | Bartunek 2017 (low dose) | | | | – | | Bartolucci 2017 | | | | – | |
|  | | Bartunek 2017 (mid dose) | | | |  | |  | | | |  | |
|  | | Bartunek 2017 (high dose) | | | |  | |  | | | |  | |
|  | | Choudhury 2017 (ICI) | | | |  | |  | | | |  | |
|  | | Choudhury 2017 (TESI) | | | |  | |  | | | |  | |
|  | | Heldman 2014 (BMMNC) | | | |  | |  | | | |  | |
|  | | Heldman 2014 (BMMSC) | | | |  | |  | | | |  | |
|  | | Henry 2014 (ischemic) | | | |  | |  | | | |  | |
|  | | Henry 2014 (non-ischemic) | | | |  | |  | | | |  | |
|  | | Hu 2011 | | | |  | |  | | | |  | |
|  | | Martino 2015 | | | |  | |  | | | |  | |
|  | | Patel 2015 (ischemic) | | | |  | |  | | | |  | |
|  | | Patel 2015 (non-ischemic) | | | |  | |  | | | |  | |
|  | | Patel 2016 | | | |  | |  | | | |  | |
|  | | Patila 2014 | | | |  | |  | | | |  | |
|  | | Perin 2015 (25 mio) | | | |  | |  | | | |  | |
|  | | Perin 2015 (75 mio) | | | |  | |  | | | |  | |
|  | | Perin 2015 (150 mio) | | | |  | |  | | | |  | |
|  | | Pokushalov 2010 | | | |  | |  | | | |  | |
|  | | Qi 2018 | | | |  | |  | | | |  | |
|  | | Sant'Anna 2014 | | | |  | |  | | | |  | |
|  | | Trifunovic 2015 | | | |  | |  | | | |  | |
|  | | Vrtovec 2011 | | | |  | |  | | | |  | |
|  | | Xiao 2017 (BMMNC) | | | |  | |  | | | |  | |
|  | | Xiao 2017 (BMMSC) | | | |  | |  | | | |  | |
|  | |  | | | |  | |  | | | |  | |
| **Fig 6F** | | | | | | | | | | | | | |
| ***muscle-derived*** | | ***BM-derived*** | | | | ***AT-derived*** | | ***perinatal*** | | | | ***cardiac-derived*** | |
| Brickwedel 2013 | | Ang 2008 (ICI) | | | | Henry 2017 | | Bartolucci 2017 | | | | Makkar 2020 | |
|  | | Ang 2008 (IMI) | | | |  | |  | | | |  | |
|  | | Assmus 2013 (low dose) | | | |  | |  | | | |  | |
|  | | Assmus 2013 (high dose) | | | |  | |  | | | |  | |
|  | | Bartunek 2013 | | | |  | |  | | | |  | |
|  | | Bartunek 2017 (low dose) | | | |  | |  | | | |  | |
|  | | Bartunek 2017 (mid dose) | | | |  | |  | | | |  | |
|  | | Bartunek 2017 (high dose) | | | |  | |  | | | |  | |
|  | | Frljak 2018 | | | |  | |  | | | |  | |
|  | | Henry 2014 (ischemic) | | | |  | |  | | | |  | |
|  | | Henry 2014 (non-ischemic) | | | |  | |  | | | |  | |
|  | | Martino 2015 | | | |  | |  | | | |  | |
|  | | Mathiasen 2015 | | | |  | |  | | | |  | |
|  | | Maureira 2012 | | | |  | |  | | | |  | |
|  | | Nasseri 2014 | | | |  | |  | | | |  | |
|  | | Noiseux 2016 | | | |  | |  | | | |  | |
|  | | Patel 2005 | | | |  | |  | | | |  | |
|  | | Patel 2016 | | | |  | |  | | | |  | |
|  | | Perin 2011 | | | |  | |  | | | |  | |
|  | | Perin 2012a | | | |  | |  | | | |  | |
|  | | Perin 2012b | | | |  | |  | | | |  | |
|  | | Perin 2015 (25 mio) | | | |  | |  | | | |  | |
|  | | Perin 2015 (75 mio) | | | |  | |  | | | |  | |
|  | | Perin 2015 (150 mio) | | | |  | |  | | | |  | |
|  | | Pokushalov 2010 | | | |  | |  | | | |  | |
|  | | Sant'Anna 2014 | | | |  | |  | | | |  | |
|  | | Santoso 2014 | | | |  | |  | | | |  | |
|  | | Steinhoff 2017 | | | |  | |  | | | |  | |
|  | | Vrtovec 2011 | | | |  | |  | | | |  | |
|  | |  | | | |  | |  | | | |  | |
| **Fig 6G** | | | | | | | | | | | | | |
| ***muscle-derived*** | | ***BM derived*** | | | | ***AT derived*** | | ***perinatal*** | | | | ***cardiac-derived*** | |
| Brickwedel 2013 | | Bartunek 2017 (low dose) | | | | – | | Bartolucci 2017 | | | | – | |
| Dib 2009 | | Bartunek 2017 (mid dose) | | | |  | |  | | | |  | |
|  | | Bartunek 2017 (high dose) | | | |  | |  | | | |  | |
|  | | Choudhury 2017 (ICI) | | | |  | |  | | | |  | |
|  | | Choudhury 2017 (TESI) | | | |  | |  | | | |  | |
|  | | Heldman 2014 (BMMNC) | | | |  | |  | | | |  | |
|  | | Heldman 2014 (BMMSC) | | | |  | |  | | | |  | |
|  | | Henry 2014 (ischemic) | | | |  | |  | | | |  | |
|  | | Henry 2014 (non-ischemic) | | | |  | |  | | | |  | |
|  | | Hu 2011 | | | |  | |  | | | |  | |
|  | | Martino 2015 | | | |  | |  | | | |  | |
|  | | Patel 2016 | | | |  | |  | | | |  | |
|  | | Perin 2015 (25 mio) | | | |  | |  | | | |  | |
|  | | Perin 2015 (75 mio) | | | |  | |  | | | |  | |
|  | | Perin 2015 (150 mio) | | | |  | |  | | | |  | |
|  | | Pokushalov 2010 | | | |  | |  | | | |  | |
|  | | Sant'Anna 2014 | | | |  | |  | | | |  | |
|  | | Vrtovec 2011 | | | |  | |  | | | |  | |
|  | |  | | | |  | |  | | | |  | |
| **Fig 6H** | | | | | | | | | | | | | |
| ***muscle-derived*** | | ***BM-derived*** | | | | ***AT-derived*** | | ***perinatal*** | | | | ***cardiac-derived*** | |
| Dib 2009 | | Assmus 2013 (low dose) | | | | Perin 2014 | | Bartolucci 2017 | | | | – | |
| Duckers 2011 | | Assmus 2013 (high dose) | | | |  | |  | | | |  | |
|  | | Choudhury 2017 (ICI) | | | |  | |  | | | |  | |
|  | | Choudhury 2017 (TESI) | | | |  | |  | | | |  | |
|  | | Mathiasen 2015 | | | |  | |  | | | |  | |
|  | | Nasseri 2014 | | | |  | |  | | | |  | |
|  | | Patel 2005 | | | |  | |  | | | |  | |
|  | | Perin 2011 | | | |  | |  | | | |  | |
|  | | Perin 2012a | | | |  | |  | | | |  | |
|  | | Pokushalov 2010 | | | |  | |  | | | |  | |
|  | | Sant'Anna 2014 | | | |  | |  | | | |  | |
|  | | Trifunovic 2015 | | | |  | |  | | | |  | |
|  | | Xiao 2017 (BMMNC) | | | |  | |  | | | |  | |
|  | | Xiao 2017 (BMMSC) | | | |  | |  | | | |  | |
|  | | Zhao 2008 | | | |  | |  | | | |  | |
|  | |  | | | |  | |  | | | |  | |
| **Fig 6I** | | | | | | | | | | | | | |
| ***muscle-derived*** | | ***BM-derived*** | | | | ***AT-derived*** | | ***perinatal*** | | | | ***cardiac-derived*** | |
| Dib 2009 | | Choudhury 2017 (ICI) | | | | Perin 2014 | | Bartolucci 2017 | | | | – | |
|  | | Choudhury 2017 (TESI) | | | |  | |  | | | |  | |
|  | | Pokushalov 2010 | | | |  | |  | | | |  | |
|  | | Sant'Anna 2014 | | | |  | |  | | | |  | |
|  | | Seth 2006 | | | |  | |  | | | |  | |
|  | | Trifunovic 2015 | | | |  | |  | | | |  | |
|  | | Xiao 2017 (BMMNC) | | | |  | |  | | | |  | |
|  | | Xiao 2017 (BMMSC) | | | |  | |  | | | |  | |
|  | |  | | | |  | |  | | | |  | |
| **Fig 7A** | | | | | | | | | | | | | |
| ***BMMNCs*** | ***CD34+*** | | ***CD133+*** | | ***ALDH+*** | | ***BMMSCs*** | | ***cardiopoietic*** | | ***STRO-3+ MPCs*** | | ***Ixmylocel-T*** |
| Ang 2008 | Patel 2005 | | Nasseri 2014 | | Perin 2012a | | Mathiasen 2015 | | – | | – | | Henry 2014 (ischemic) |
| Assumus 2013 | Vrtovec 2011 | | Noiseux 2016 | |  | | Xiao 2017 (BMMSC) | |  | |  | |  |
| Frljak 2018 |  | |  | |  | |  | |  | |  | |  |
| Hu 2011 |  | |  | |  | |  | |  | |  | |  |
| Martino 2015 |  | |  | |  | |  | |  | |  | |  |
| Maureira 2012 |  | |  | |  | |  | |  | |  | |  |
| Perin 2011 |  | |  | |  | |  | |  | |  | |  |
| Perin 2012b |  | |  | |  | |  | |  | |  | |  |
| Pokushalov 2010 |  | |  | |  | |  | |  | |  | |  |
| Sant'Anna 2014 |  | |  | |  | |  | |  | |  | |  |
| Santoso 2014 |  | |  | |  | |  | |  | |  | |  |
| Seth 2006 |  | |  | |  | |  | |  | |  | |  |
| Wang 2015 |  | |  | |  | |  | |  | |  | |  |
| Xiao 2017 (BMMNC) |  | |  | |  | |  | |  | |  | |  |
| Zhao 2008 |  | |  | |  | |  | |  | |  | |  |
|  |  | |  | |  | |  | |  | |  | |  |
| **Fig 7B** | | | | | | | | | | | | | |
| ***BMMNCs*** | ***CD34+*** | | ***CD133+*** | | ***ALDH+*** | | ***BMMSCs*** | | ***cardiopoietic*** | | ***STRO-3+ MPCs*** | | ***Ixmylocel-T*** |
| Choudhury 2017 (ICI) | Vrtovec 2011 | | Steinhoff 2017 | | – | | Heldman 2014 (BMMSC) | | Bartunek 2017 | | – | | Henry 2014 (ischemic) |
| Choudhury 2017 (TESI) |  | |  | |  | | Xiao 2017 (BMMSC) | |  | |  | | Henry 2014 (non-ischemic) |
| Heldman 2014 (BMMNC) |  | |  | |  | |  | |  | |  | | Patel 2016 |
| Hu 2011 |  | |  | |  | |  | |  | |  | |  |
| Martino 2015 |  | |  | |  | |  | |  | |  | |  |
| Patel 2015 (ischemic) |  | |  | |  | |  | |  | |  | |  |
| Patel 2015 (nonischemic) |  | |  | |  | |  | |  | |  | |  |
| Patila 2014 |  | |  | |  | |  | |  | |  | |  |
| Perin 2011 |  | |  | |  | |  | |  | |  | |  |
| Pokushalov 2010 |  | |  | |  | |  | |  | |  | |  |
| Qi 2018 |  | |  | |  | |  | |  | |  | |  |
| Sant'Anna 2014 |  | |  | |  | |  | |  | |  | |  |
| Xiao 2017 (BMMNC) |  | |  | |  | |  | |  | |  | |  |
|  |  | |  | |  | |  | |  | |  | |  |
| **Fig 7C** | | | | | | | | | | | | | |
| ***BMMNCs*** | ***CD34+*** | | ***CD133+*** | | ***ALDH+*** | | ***BMMSCs*** | | ***cardiopoietic*** | | ***STRO-3+ MPCs*** | | ***Ixmylocel-T*** |
| Patila 2014 | Vrtovec 2011 | | Nasseri 2014 | |  | |  | | Bartunek 2013 | | Perin 2015 (25 mio) | | – |
| Pokushalov 2010 |  | | Steinhoff 2017 | |  | |  | |  | | Perin 2015 (75 mio) | |  |
| Santoso 2014 |  | |  | |  | |  | |  | | Perin 2015 (150 mio) | |  |
| Seth 2006 |  | |  | |  | |  | |  | |  | |  |
| Trifunovic 2015 |  | |  | |  | |  | |  | |  | |  |
|  |  | |  | |  | |  | |  | |  | |  |
| **Fig 7D** | | | | | | | | | | | | | |
| ***BMMNCs*** | ***CD34+*** | | ***CD133+*** | | ***ALDH+*** | | ***BMMSCs*** | | ***cardiopoietic*** | | ***STRO-3+ MPCs*** | | ***Ixmylocel-T*** |
| Ang 2008 (ICI) | Patel 2005 | | Nasseri 2014 | | Perin 2012a | | Mathiasen 2015 | | Bartunek 2013 | | Perin 2015 (25 mio) | | Henry 2014 (ischemic) |
| Ang 2008 (IMI) | Vrtovec 2011 | | Noiseux 2016 | |  | | Xiao 2017 (BMMSC) | | Bartunek 2017 (low dose) | | Perin 2015 (75 mio) | | Henry 2014 (non-ischemic) |
| Assmus 2013 (low dose) |  | | Steinhoff 2017 | |  | |  | | Bartunek 2017 (mid dose) | | Perin 2015 (150 mio) | | Patel 2016 |
| Assmus 2013 (high dose) |  | |  | |  | |  | | Bartunek 2017 (high dose) | |  | |  |
| Frljak 2018 |  | |  | |  | |  | |  | |  | |  |
| Martino 2015 |  | |  | |  | |  | |  | |  | |  |
| Maureira 2012 |  | |  | |  | |  | |  | |  | |  |
| Patel 2015 (ischemic) |  | |  | |  | |  | |  | |  | |  |
| Patel 2015 (non-ischemic) |  | |  | |  | |  | |  | |  | |  |
| Perin 2011 |  | |  | |  | |  | |  | |  | |  |
| Perin 2012b |  | |  | |  | |  | |  | |  | |  |
| Pokushalov 2010 |  | |  | |  | |  | |  | |  | |  |
| Sant'Anna 2014 |  | |  | |  | |  | |  | |  | |  |
| Santoso 2014 |  | |  | |  | |  | |  | |  | |  |
| Seth 2006 |  | |  | |  | |  | |  | |  | |  |
| Trifunovic 2015 |  | |  | |  | |  | |  | |  | |  |
| Wang 2015 |  | |  | |  | |  | |  | |  | |  |
| Xiao 2017 (BMMNC) |  | |  | |  | |  | |  | |  | |  |
| Zhao 2008 |  | |  | |  | |  | |  | |  | |  |
|  |  | |  | |  | |  | |  | |  | |  |
| **Fig 7E** | | | | | | | | | | | | | |
| ***BMMNCs*** | ***CD34+*** | | ***CD133+*** | | ***ALDH+*** | | ***BMMSCs*** | | ***cardiopoietic*** | | ***STRO-3+ MPCs*** | | ***Ixmylocel-T*** |
| Choudhury 2017 (ICI) | Vrtovec 2011 | | – | | – | | Heldman 2014 (BMMSC) | | Bartunek 2017 (low dose) | | Perin 2015 (25 mio) | | Henry 2014 (ischemic) |
| Choudhury 2017 (TESI) |  | |  | |  | | Xiao 2017 (BMMSC) | | Bartunek 2017 (mid dose) | | Perin 2015 (75 mio) | | Henry 2014 (non-ischemic) |
| Heldman 2014 (BMMNC) |  | |  | |  | |  | | Bartunek 2017 (high dose) | | Perin 2015 (150 mio) | | Patel 2016 |
| Hu 2011 |  | |  | |  | |  | |  | |  | |  |
| Martino 2015 |  | |  | |  | |  | |  | |  | |  |
| Patel 2015 (ischemic) |  | |  | |  | |  | |  | |  | |  |
| Patel 2015 (non-ischemic) |  | |  | |  | |  | |  | |  | |  |
| Patila 2014 |  | |  | |  | |  | |  | |  | |  |
| Pokushalov 2010 |  | |  | |  | |  | |  | |  | |  |
| Qi 2018 |  | |  | |  | |  | |  | |  | |  |
| Sant'Anna 2014 |  | |  | |  | |  | |  | |  | |  |
| Trifunovic 2015 |  | |  | |  | |  | |  | |  | |  |
| Xiao 2017 (BMMNC) |  | |  | |  | |  | |  | |  | |  |
|  |  | |  | |  | |  | |  | |  | |  |
| **Fig 7F** | | | | | | | | | | | | | |
| ***BMMNCs*** | ***CD34+*** | | ***CD133+*** | | ***ALDH+*** | | ***BMMSCs*** | | ***cardiopoietic*** | | ***STRO-3+ MPCs*** | | ***Ixmylocel-T*** |
| Ang 2008 (ICI) | Patel 2005 | | Nasseri 2014 | | Perin 2012a | | Mathiasen 2015 | | Bartunek 2013 | | Perin 2015 (25 mio) | | Henry 2014 (ischemic) |
| Ang 2008 (IMI) | Vrtovec 2011 | | Noiseux 2016 | |  | |  | | Bartunek 2017 (low dose) | | Perin 2015 (75 mio) | | Henry 2014 (non-ischemic) |
| Assmus 2013 (low dose) |  | | Steinhoff 2017 | |  | |  | | Bartunek 2017 (mid dose) | | Perin 2015 (150 mio) | | Patel 2016 |
| Assmus 2013 (high dose) |  | |  | |  | |  | | Bartunek 2017 (high dose) | |  | |  |
| Frljak 2018 |  | |  | |  | |  | |  | |  | |  |
| Martino 2015 |  | |  | |  | |  | |  | |  | |  |
| Maureira 2012 |  | |  | |  | |  | |  | |  | |  |
| Perin 2011 |  | |  | |  | |  | |  | |  | |  |
| Perin 2012b |  | |  | |  | |  | |  | |  | |  |
| Pokushalov 2010 |  | |  | |  | |  | |  | |  | |  |
| Sant'Anna 2014 |  | |  | |  | |  | |  | |  | |  |
| Santoso 2014 |  | |  | |  | |  | |  | |  | |  |
|  |  | |  | |  | |  | |  | |  | |  |
| **Fig 7G** | | | | | | | | | | | | | |
| ***BMMNCs*** | ***CD34+*** | | ***CD133+*** | | ***ALDH+*** | | ***BMMSCs*** | | ***cardiopoietic*** | | ***STRO-3+ MPCs*** | | ***Ixmylocel-T*** |
| Choudhury 2017 (ICI) | Vrtovec 2011 | | – | | – | | Heldman 2014 BMMSC | | Bartunek 2017 (low dose) | | Perin 2015 (25 mio) | | Henry 2014 (ischemic) |
| Choudhury 2017 (TESI) |  | |  | |  | |  | | Bartunek 2017 (mid dose) | | Perin 2015 (75 mio) | | Henry 2014 (non-ischemic) |
| Heldman 2014 (BMMNC) |  | |  | |  | |  | | Bartunek 2017 (high dose) | | Perin 2015 (150 mio) | | Patel 2016 |
| Hu 2011 |  | |  | |  | |  | |  | |  | |  |
| Martino 2015 |  | |  | |  | |  | |  | |  | |  |
| Pokushalov 2010 |  | |  | |  | |  | |  | |  | |  |
| Sant'Anna 2014 |  | |  | |  | |  | |  | |  | |  |
|  |  | |  | |  | |  | |  | |  | |  |
| **Fig 7H** | | | | | | | | | | | | | |
| ***BMMNCs*** | ***CD34+*** | | ***CD133+*** | | ***ALDH+*** | | ***BMMSCs*** | | ***cardiopoietic*** | | ***STRO-3+ MPCs*** | | ***Ixmylocel-T*** |
| Assmus 2013 (low dose) | Patel 2005 | | Nasseri 2014 | | Perin 2012a | | Mathiasen 2015 | | – | | – | | – |
| Assmus 2013 (high dose) |  | |  | |  | | Xiao 2017 (BMMSC) | |  | |  | |  |
| Choudhury 2017 (ICI) |  | |  | |  | |  | |  | |  | |  |
| Choudhury 2017 (TESI) |  | |  | |  | |  | |  | |  | |  |
| Perin 2011 |  | |  | |  | |  | |  | |  | |  |
| Pokushalov 2010 |  | |  | |  | |  | |  | |  | |  |
| Sant'Anna 2014 |  | |  | |  | |  | |  | |  | |  |
| Trifunovic 2015 |  | |  | |  | |  | |  | |  | |  |
| Xiao 2017 (BMMNC) |  | |  | |  | |  | |  | |  | |  |
| Zhao 2008 |  | |  | |  | |  | |  | |  | |  |
|  |  | |  | |  | |  | |  | |  | |  |
| **Fig 7I** | | | | | | | | | | | | | |
| ***BMMNCs*** | ***CD34+*** | | ***CD133+*** | | ***ALDH+*** | | ***BMMSCs*** | | ***cardiopoietic*** | | ***STRO-3+ MPCs*** | | ***Ixmylocel-Ts*** |
| Choudhury 2017 (ICI) | – | | – | | – | | Xiao 2017 (BMMSC) | | – | | – | | – |
| Choudhury 2017 (TESI) |  | |  | |  | |  | |  | |  | |  |
| Pokushalov 2010 |  | |  | |  | |  | |  | |  | |  |
| Sant'Anna 2014 |  | |  | |  | |  | |  | |  | |  |
| Seth 2006 |  | |  | |  | |  | |  | |  | |  |
| Trifunovic 2015 |  | |  | |  | |  | |  | |  | |  |
| Xiao 2017 (BMMNC) |  | |  | |  | |  | |  | |  | |  |
|  |  | |  | |  | |  | |  | |  | |  |
| **Fig 8A** | | | | | | | | | | | | | |
| ***ischemic HF*** | | | | ***non-ischemic HF*** | | | | | | ***both*** | | | |
| Ang 2008 | | | | Frljak 2018 | | | | | | – | | | |
| Assumus 2013 | | | | Martino 2015 | | | | | |  | | | |
| Henry 2014 (ischemic) | | | | Sant'Anna 2014 | | | | | |  | | | |
| Hu 2011 | | | | Seth 2006 | | | | | |  | | | |
| Mathiasen 2015 | | | | Vrtovec 2011 | | | | | |  | | | |
| Maureira 2012 | | | | Xiao 2017 (BMMNC) | | | | | |  | | | |
| Nasseri 2014 | | | | Xiao 2017 (BMMSC) | | | | | |  | | | |
| Noiseux 2016 | | | |  | | | | | |  | | | |
| Patel 2005 | | | |  | | | | | |  | | | |
| Perin 2011 | | | |  | | | | | |  | | | |
| Perin 2012a | | | |  | | | | | |  | | | |
| Perin 2012b | | | |  | | | | | |  | | | |
| Pokushalov 2010 | | | |  | | | | | |  | | | |
| Santoso 2014 | | | |  | | | | | |  | | | |
| Wang 2015 | | | |  | | | | | |  | | | |
| Zhao 2008 | | | |  | | | | | |  | | | |
|  | | | |  | | | | | |  | | | |
| **Fig 8B** | | | | | | | | | | | | | |
| ***ischemic HF*** | | | | ***non-ischemic HF*** | | | | | | ***both*** | | | |
| Bartunek 2017 | | | | Martino 2015 | | | | | | – | | | |
| Choudhury 2017 (ICI) | | | | Patel 2015 (non-ischemic) | | | | | |  | | | |
| Choudhury 2017 (TESI) | | | | Sant'Anna 2014 | | | | | |  | | | |
| Heldman 2014 (BMMNC) | | | | Vrtovec 2011 | | | | | |  | | | |
| Heldman 2014 (BMMSC) | | | | Xiao 2017 (BMMNC) | | | | | |  | | | |
| Henry 2014 (ischemic) | | | | Xiao 2017 (BMMSC) | | | | | |  | | | |
| Hu 2011 | | | |  | | | | | |  | | | |
| Patel 2015 (ischemic) | | | |  | | | | | |  | | | |
| Patel 2016 | | | |  | | | | | |  | | | |
| Patila 2014 | | | |  | | | | | |  | | | |
| Perin 2011 | | | |  | | | | | |  | | | |
| Pokushalov 2010 | | | |  | | | | | |  | | | |
| Qi 2018 | | | |  | | | | | |  | | | |
| Steinhoff 2017 | | | |  | | | | | |  | | | |
|  | | | |  | | | | | |  | | | |
| **Fig 8C** | | | | | | | | | | | | | |
| ***ischemic HF*** | | | | ***non-ischemic HF*** | | | | | | ***both*** | | | |
| Bartunek 2013 | | | | Seth 2006 | | | | | | Perin 2015 (25 mio) | | | |
| Nasseri 2014 | | | | Vrtovec 2011 | | | | | | Perin 2015 (75 mio) | | | |
| Patila 2014 | | | |  | | | | | | Perin 2015 (150 mio) | | | |
| Pokushalov 2010 | | | |  | | | | | |  | | | |
| Santoso 2014 | | | |  | | | | | |  | | | |
| Steinhoff 2017 | | | |  | | | | | |  | | | |
| Trifunovic 2015 | | | |  | | | | | |  | | | |
|  | | | |  | | | | | |  | | | |
| **Fig 8D** | | | | | | | | | | | | | |
| ***ischemic HF*** | | | | ***non-ischemic HF*** | | | | | | ***both*** | | | |
| Ang 2008 (ICI) | | | | Frljak 2018 | | | | | | Perin 2015 (25 mio) | | | |
| Ang 2008 (IMI) | | | | Henry 2014 (non-ischemic) | | | | | | Perin 2015 (75 mio) | | | |
| Assmus 2013 (low dose) | | | | Martino 2015 | | | | | | Perin 2015 (150 mio) | | | |
| Assmus 2013 (high dose) | | | | Patel 2015 (non-ischemic) | | | | | |  | | | |
| Bartunek 2013 | | | | Sant'Anna 2014 | | | | | |  | | | |
| Bartunek 2017 (low dose) | | | | Seth 2006 | | | | | |  | | | |
| Bartunek 2017 (mid dose) | | | | Vrtovec 2011 | | | | | |  | | | |
| Bartunek 2017 (high dose) | | | | Xiao 2017 (BMMNC) | | | | | |  | | | |
| Henry 2014 (ischemic) | | | | Xiao 2017 (BMMSC) | | | | | |  | | | |
| Mathiasen 2015 | | | |  | | | | | |  | | | |
| Maureira 2012 | | | |  | | | | | |  | | | |
| Nasseri 2014 | | | |  | | | | | |  | | | |
| Noiseux 2016 | | | |  | | | | | |  | | | |
| Patel 2005 | | | |  | | | | | |  | | | |
| Patel 2015 (ischemic) | | | |  | | | | | |  | | | |
| Patel 2016 | | | |  | | | | | |  | | | |
| Perin 2011 | | | |  | | | | | |  | | | |
| Perin 2012a | | | |  | | | | | |  | | | |
| Perin 2012b | | | |  | | | | | |  | | | |
| Pokushalov 2010 | | | |  | | | | | |  | | | |
| Santoso 2014 | | | |  | | | | | |  | | | |
| Steinhoff 2017 | | | |  | | | | | |  | | | |
| Trifunovic 2015 | | | |  | | | | | |  | | | |
| Wang 2015 | | | |  | | | | | |  | | | |
| Zhao 2008 | | | |  | | | | | |  | | | |
|  | | | |  | | | | | |  | | | |
| **Fig 8E** | | | | | | | | | | | | | |
| ***ischemic HF*** | | | | ***non-ischemic HF*** | | | | | | ***both*** | | | |
| Bartunek 2017 (low dose) | | | | Henry 2014 (non-ischemic) | | | | | | Perin 2015 (25 mio) | | | |
| Bartunek 2017 (mid dose) | | | | Martino 2015 | | | | | | Perin 2015 (75 mio) | | | |
| Bartunek 2017 (high dose) | | | | Patel 2015 (non-ischemic) | | | | | | Perin 2015 (150 mio) | | | |
| Choudhury 2017 (ICI) | | | | Sant'Anna 2014 | | | | | |  | | | |
| Choudhury 2017 (TESI) | | | | Vrtovec 2011 | | | | | |  | | | |
| Heldman 2014 (BMMNC) | | | | Xiao 2017 (BMMNC) | | | | | |  | | | |
| Heldman 2014 (BMMSC) | | | | Xiao 2017 (BMMSC) | | | | | |  | | | |
| Henry 2014 (ischemic) | | | |  | | | | | |  | | | |
| Hu 2011 | | | |  | | | | | |  | | | |
| Patel 2015 (ischemic) | | | |  | | | | | |  | | | |
| Patel 2016 | | | |  | | | | | |  | | | |
| Patila 2014 | | | |  | | | | | |  | | | |
| Pokushalov 2010 | | | |  | | | | | |  | | | |
| Qi 2018 | | | |  | | | | | |  | | | |
| Trifunovic 2015 | | | |  | | | | | |  | | | |
|  | | | |  | | | | | |  | | | |
| **Fig 8F** | | | | | | | | | | | | | |
| ***ischemic HF*** | | | | ***non-ischemic HF*** | | | | | | ***both*** | | | |
| Ang 2008 (ICI) | | | | Frljak 2018 | | | | | | Perin 2015 (25 mio) | | | |
| Ang 2008 (IMI) | | | | Henry 2014 (non-ischemic) | | | | | | Perin 2015 (75 mio) | | | |
| Assmus 2013 (low dose) | | | | Martino 2015 | | | | | | Perin 2015 (150 mio) | | | |
| Assmus 2013 (high dose) | | | | Sant'Anna 2014 | | | | | |  | | | |
| Bartunek 2013 | | | | Vrtovec 2011 | | | | | |  | | | |
| Bartunek 2017 (low dose) | | | |  | | | | | |  | | | |
| Bartunek 2017 (mid dose) | | | |  | | | | | |  | | | |
| Bartunek 2017 (high dose) | | | |  | | | | | |  | | | |
| Henry 2014 (ischemic) | | | |  | | | | | |  | | | |
| Mathiasen 2015 | | | |  | | | | | |  | | | |
| Maureira 2012 | | | |  | | | | | |  | | | |
| Nasseri 2014 | | | |  | | | | | |  | | | |
| Noiseux 2016 | | | |  | | | | | |  | | | |
| Patel 2005 | | | |  | | | | | |  | | | |
| Patel 2016 | | | |  | | | | | |  | | | |
| Perin 2011 | | | |  | | | | | |  | | | |
| Perin 2012a | | | |  | | | | | |  | | | |
| Perin 2012b | | | |  | | | | | |  | | | |
| Pokushalov 2010 | | | |  | | | | | |  | | | |
| Santoso 2014 | | | |  | | | | | |  | | | |
| Steinhoff 2017 | | | |  | | | | | |  | | | |
|  | | | |  | | | | | |  | | | |
| **Fig 8G** | | | | | | | | | | | | | |
| ***ischemic HF*** | | | | ***non-ischemic HF*** | | | | | | ***both*** | | | |
| Bartunek 2017 (low dose) | | | | Henry 2014 (non-ischemic) | | | | | | Perin 2015 (25 mio) | | | |
| Bartunek 2017 (mid dose) | | | | Martino 2015 | | | | | | Perin 2015 (75 mio) | | | |
| Bartunek 2017 (high dose) | | | | Sant'Anna 2014 | | | | | | Perin 2015 (150 mio) | | | |
| Choudhury 2017 (ICI) | | | | Vrtovec 2011 | | | | | |  | | | |
| Choudhury 2017 (TESI) | | | |  | | | | | |  | | | |
| Heldman 2014 (BMMNC) | | | |  | | | | | |  | | | |
| Heldman 2014 (BMMSC) | | | |  | | | | | |  | | | |
| Henry 2014 (ischemic) | | | |  | | | | | |  | | | |
| Hu 2011 | | | |  | | | | | |  | | | |
| Patel 2016 | | | |  | | | | | |  | | | |
| Pokushalov 2010 | | | |  | | | | | |  | | | |
|  | | | |  | | | | | |  | | | |
| **Fig 8H** | | | | | | | | | | | | | |
| ***ischemic HF*** | | | | ***non-ischemic HF*** | | | | | | ***both*** | | | |
| Assmus 2013 (low dose) | | | | Sant'Anna 2014 | | | | | | – | | | |
| Assmus 2013 (high dose) | | | | Xiao 2017 (BMMNC) | | | | | |  | | | |
| Choudhury 2017 (ICI) | | | | Xiao 2017 (BMMSC) | | | | | |  | | | |
| Choudhury 2017 (TESI) | | | |  | | | | | |  | | | |
| Mathiasen 2015 | | | |  | | | | | |  | | | |
| Nasseri 2014 | | | |  | | | | | |  | | | |
| Patel 2005 | | | |  | | | | | |  | | | |
| Perin 2011 | | | |  | | | | | |  | | | |
| Perin 2012a | | | |  | | | | | |  | | | |
| Pokushalov 2010 | | | |  | | | | | |  | | | |
| Trifunovic 2015 | | | |  | | | | | |  | | | |
| Zhao 2008 | | | |  | | | | | |  | | | |
|  | | | |  | | | | | |  | | | |
| **Fig 8I** | | | | | | | | | | | | | |
| ***ischemic HF*** | | | | ***non-ischemic HF*** | | | | | | ***both*** | | | |
| Choudhury 2017 (ICI) | | | | Sant'Anna 2014 | | | | | | – | | | |
| Choudhury 2017 (TESI) | | | | Seth 2006 | | | | | |  | | | |
| Pokushalov 2010 | | | | Xiao 2017 (BMMNC) | | | | | |  | | | |
| Trifunovic 2015 | | | | Xiao 2017 (BMMSC) | | | | | |  | | | |
|  | | | |  | | | | | |  | | | |
| **Fig 9A** | | | | | | | | | | | | | |
| ***CABG+IMI*** | | ***CABG+ICI*** | | | | ***TESI*** | | ***IMI*** | | | | ***ICI*** | |
| Ang 2008 IMI | | Ang 2008 ICI | | | | Frljak 2018 | | Henry 2014 IMI | | | | Assumus 2013 | |
| Maureira 2012 | | Hu 2011 | | | | Mathiasen 2015 | | Sant'Anna 2014 | | | | Martino 2015 | |
| Nasseri 2014 | |  | | | | Perin 2011 | |  | | | | Seth 2006 | |
| Noiseux 2016 | |  | | | | Perin 2012a | |  | | | | Vrtovec 2011 | |
| Patel 2005 | |  | | | | Perin 2012b | |  | | | | Xiao 2017 BMMNC | |
| Wang 2015 | |  | | | | Pokushalov 2010 | |  | | | | Xiao 2017 BMMSC | |
| Zhao 2008 | |  | | | | Santoso 2014 | |  | | | |  | |
|  | |  | | | |  | |  | | | |  | |
| **Fig 9B** | | | | | | | | | | | | | |
| ***CABG+IMI*** | | ***CABG+ICI*** | | | | ***TESI*** | | ***IMI*** | | | | ***ICI*** | |
| Patila 2014 | | Hu 2011 | | | | Bartunek 2017 | | Sant'Anna 2014 | | | | Choudhury 2017 (ICI) | |
| Steinhoff 2017 | |  | | | | Choudhury 2017 (TESI) | |  | | | | Martino 2015 | |
|  | |  | | | | Heldman 2014 (BMMNC) | |  | | | | Patel 2015 (ischemic) | |
|  | |  | | | | Heldman 2014 (BMMSC) | |  | | | | Patel 2015 (non-ischemic) | |
|  | |  | | | | Henry 2014 TESI | |  | | | | Qi 2018 | |
|  | |  | | | | Patel 2016 | |  | | | | Vrtovec 2011 | |
|  | |  | | | | Perin 2011 | |  | | | | Xiao 2017 (BMMNC) | |
|  | |  | | | | Pokushalov 2010 | |  | | | | Xiao 2017 (BMMSC) | |
|  | |  | | | |  | |  | | | |  | |
| **Fig 9C** | | | | | | | | | | | | | |
| ***CABG+IMI*** | | ***CABG+ICI*** | | | | ***TESI*** | | ***IMI*** | | | | ***ICI*** | |
| Nasseri 2014 | | – | | | | Bartunek 2013 | | – | | | | Seth 2006 | |
| Patila 2014 | |  | | | | Perin 2015 (25 mio) | |  | | | | Vrtovec 2011 | |
| Steinhoff 2017 | |  | | | | Perin 2015 (75 mio) | |  | | | |  | |
| Trifunovic 2015 | |  | | | | Perin 2015 (150 mio) | |  | | | |  | |
|  | |  | | | | Pokushalov 2010 | |  | | | |  | |
|  | |  | | | | Santoso 2014 | |  | | | |  | |
|  | |  | | | |  | |  | | | |  | |
| **Fig 9D** | | | | | | | | | | | | | |
| ***CABG+IMI*** | | ***CABG+ICI*** | | | | ***TESI*** | | ***IMI*** | | | | ***ICI*** | |
| Ang 2008 (IMI) | | Ang 2008 ICI | | | | Bartunek 2013 | | Sant'Anna 2014 | | | | Assmus 2013 (low dose) | |
| Maureira 2012 | |  | | | | Bartunek 2017 (low dose) | |  | | | | Assmus 2013 (high dose) | |
| Nasseri 2014 | |  | | | | Bartunek 2017 (mid dose) | |  | | | | Martino 2015 | |
| Noiseux 2016 | |  | | | | Bartunek 2017 (high dose) | |  | | | | Patel 2015 (ischemic) | |
| Patel 2005 | |  | | | | Frljak 2018 | |  | | | | Patel 2015 (non-ischemic) | |
| Steinhoff 2017 | |  | | | | Mathiasen 2015 | |  | | | | Seth 2006 | |
| Trifunovic 2015 | |  | | | | Patel 2016 | |  | | | | Vrtovec 2011 | |
| Wang 2015 | |  | | | | Perin 2011 | |  | | | | Xiao 2017 (BMMNC) | |
| Zhao 2008 | |  | | | | Perin 2012a | |  | | | | Xiao 2017 (BMMSC) | |
|  | |  | | | | Perin 2012b | |  | | | |  | |
|  | |  | | | | Perin 2015 (25 mio) | |  | | | |  | |
|  | |  | | | | Perin 2015 (75 mio) | |  | | | |  | |
|  | |  | | | | Perin 2015 (150 mio) | |  | | | |  | |
|  | |  | | | | Pokushalov 2010 | |  | | | |  | |
|  | |  | | | | Santoso 2014 | |  | | | |  | |
|  | |  | | | |  | |  | | | |  | |
| **Fig 9E** | | | | | | | | | | | | | |
| ***CABG+IMI*** | | ***CABG+ICI*** | | | | ***TESI*** | | ***IMI*** | | | | ***ICI*** | |
| Patila 2014 | | Hu 2011 | | | | Bartunek 2017 (low dose) | | Sant'Anna 2014 | | | | Choudhury 2017 (ICI) | |
| Trifunovic 2015 | |  | | | | Bartunek 2017 (mid dose) | |  | | | | Martino 2015 | |
|  | |  | | | | Bartunek 2017 (high dose) | |  | | | | Patel 2015 (ischemic) | |
|  | |  | | | | Choudhury 2017 (TESI) | |  | | | | Patel 2015 (non-ischemic) | |
|  | |  | | | | Heldman 2014 (BMMNC) | |  | | | | Qi 2018 | |
|  | |  | | | | Heldman 2014 (BMMSC) | |  | | | | Vrtovec 2011 | |
|  | |  | | | | Patel 2016 | |  | | | | Xiao 2017 (BMMNC) | |
|  | |  | | | | Perin 2015 (25 mio) | |  | | | | Xiao 2017 (BMMSC) | |
|  | |  | | | | Perin 2015 (75 mio) | |  | | | |  | |
|  | |  | | | | Perin 2015 (150 mio) | |  | | | |  | |
|  | |  | | | | Pokushalov 2010 | |  | | | |  | |
|  | |  | | | |  | |  | | | |  | |
| **Fig 9F** | | | | | | | | | | | | | |
| ***CABG+IMI*** | | ***CABG+ICI*** | | | | ***TESI*** | | ***IMI*** | | | | ***ICI*** | |
| Ang 2008 (IMI) | | Ang 2008 ICI | | | | Bartunek 2013 | | Sant'Anna 2014 | | | | Assmus 2013 (low dose) | |
| Maureira 2012 | |  | | | | Bartunek 2017 (low dose) | |  | | | | Assmus 2013 (high dose) | |
| Nasseri 2014 | |  | | | | Bartunek 2017 (mid dose) | |  | | | | Martino 2015 | |
| Noiseux 2016 | |  | | | | Bartunek 2017 (high dose) | |  | | | | Vrtovec 2011 | |
| Patel 2005 | |  | | | | Frljak 2018 | |  | | | |  | |
| Steinhoff 2017 | |  | | | | Mathiasen 2015 | |  | | | |  | |
| Ang 2008 (IMI) | |  | | | | Patel 2016 | |  | | | |  | |
| Maureira 2012 | |  | | | | Perin 2011 | |  | | | |  | |
| Nasseri 2014 | |  | | | | Perin 2012a | |  | | | |  | |
| Noiseux 2016 | |  | | | | Perin 2012b | |  | | | |  | |
| Patel 2005 | |  | | | | Perin 2015 (25 mio) | |  | | | |  | |
| Steinhoff 2017 | |  | | | | Perin 2015 (75 mio) | |  | | | |  | |
|  | |  | | | | Perin 2015 (150 mio) | |  | | | |  | |
|  | |  | | | | Pokushalov 2010 | |  | | | |  | |
|  | |  | | | | Santoso 2014 | |  | | | |  | |
|  | |  | | | |  | |  | | | |  | |
| **Fig 9G** | | | | | | | | | | | | | |
| ***CABG+IMI*** | | ***CABG+ICI*** | | | | ***TESI*** | | ***IMI*** | | | | ***ICI*** | |
| *–* | | Hu 2011 | | | | Bartunek 2017 (low dose) | | Sant'Anna 2014 | | | | Choudhury 2017 (ICI) | |
|  | |  | | | | Bartunek 2017 (mid dose) | |  | | | | Martino 2015 | |
|  | |  | | | | Bartunek 2017 (high dose) | |  | | | | Vrtovec 2011 | |
|  | |  | | | | Choudhury 2017 (TESI) | |  | | | |  | |
|  | |  | | | | Heldman 2014 (BMMNC) | |  | | | |  | |
|  | |  | | | | Heldman 2014 (BMMSC) | |  | | | |  | |
|  | |  | | | | Patel 2016 | |  | | | |  | |
|  | |  | | | | Perin 2015 (25 mio) | |  | | | |  | |
|  | |  | | | | Perin 2015 (75 mio) | |  | | | |  | |
|  | |  | | | | Perin 2015 (150 mio) | |  | | | |  | |
|  | |  | | | | Pokushalov 2010 | |  | | | |  | |
|  | |  | | | |  | |  | | | |  | |
| **Fig 9H** | | | | | | | | | | | | | |
| ***CABG+IMI*** | | ***CABG+ICI*** | | | | ***TESI*** | | ***IMI*** | | | | ***ICI*** | |
| Nasseri 2014 | | *–* | | | | Choudhury 2017 (TESI) | | Sant'Anna 2014 | | | | Assmus 2013 (low dose) | |
| Patel 2005 | |  | | | | Mathiasen 2015 | |  | | | | Assmus 2013 (high dose) | |
| Trifunovic 2015 | |  | | | | Perin 2011 | |  | | | | Choudhury 2017 (ICI) | |
| Zhao 2008 | |  | | | | Perin 2012a | |  | | | | Xiao 2017 (BMMNC) | |
|  | |  | | | | Pokushalov 2010 | |  | | | | Xiao 2017 (BMMSC) | |
|  | |  | | | |  | |  | | | |  | |
| **Fig 9I** | | | | | | | | | | | | | |
| ***CABG+IMI*** | | ***CABG+ICI*** | | | | ***TESI*** | | ***IMI*** | | | | ***ICI*** | |
| Trifunovic 2015 | | – | | | | Choudhury 2017 (TESI) | | Sant'Anna 2014 | | | | Choudhury 2017 (ICI) | |
|  | |  | | | | Pokushalov 2010 | |  | | | | Seth 2006 | |
|  | |  | | | |  | |  | | | | Xiao 2017 (BMMNC) | |
|  | |  | | | |  | |  | | | | Xiao 2017 (BMMSC) | |
|  | |  | | | |  | |  | | | |  | |
| **Fig 12A** | | | | | | | | | | | | | |
| ***< 1 mio*** | | | ***1 to ≤ 10 mio*** | | | | ***> 10 to ≤ 100 mio*** | | | | ***> 100 mio*** | | |
| Ang 2008 | | | Assumus 2013 | | | | Frljak 2018 | | | | Vrtovec 2011 | | |
|  | | | Henry 2014 (ischemic) | | | | Mathiasen 2015 | | | | Xiao 2017 (BMMSC) | | |
|  | | | Hu 2011 | | | | Maureira 2012 | | | |  | | |
|  | | | Martino 2015 | | | | Patel 2005 | | | |  | | |
|  | | | Nasseri 2014 | | | | Zhao 2008 | | | |  | | |
|  | | | Noiseux 2016 | | | |  | | | |  | | |
|  | | | Perin 2011 | | | |  | | | |  | | |
|  | | | Perin 2012a | | | |  | | | |  | | |
|  | | | Perin 2012b | | | |  | | | |  | | |
|  | | | Pokushalov 2010 | | | |  | | | |  | | |
|  | | | Sant'Anna 2014 | | | |  | | | |  | | |
|  | | | Santoso 2014 | | | |  | | | |  | | |
|  | | | Seth 2006 | | | |  | | | |  | | |
|  | | | Wang 2015 | | | |  | | | |  | | |
|  | | | Xiao 2017 (BMMNC) | | | |  | | | |  | | |
|  | | |  | | | |  | | | |  | | |
| **Fig 12B** | | | | | | | | | | | | | |
| ***< 1 mio*** | | | ***1 to ≤ 10 mio*** | | | | ***> 10 to ≤ 100 mio*** | | | | ***> 100 mio*** | | |
| – | | | Choudhury 2017 (ICI) | | | | Patel 2015 (ischemic) | | | | Bartunek 2017 | | |
|  | | | Choudhury 2017 (TESI) | | | | Patel 2015 (non-ischemic) | | | | Heldman 2014 (BMMSC) | | |
|  | | | Heldman 2014 (BMMNC) | | | | Patila 2014 | | | | Vrtovec 2011 | | |
|  | | | Henry 2014 (ischemic) | | | |  | | | | Xiao 2017 (BMMSC) | | |
|  | | | Henry 2014 (non-ischemic) | | | |  | | | |  | | |
|  | | | Hu 2011 | | | |  | | | |  | | |
|  | | | Martino 2015 | | | |  | | | |  | | |
|  | | | Patel 2016 | | | |  | | | |  | | |
|  | | | Perin 2011 | | | |  | | | |  | | |
|  | | | Pokushalov 2010 | | | |  | | | |  | | |
|  | | | Qi 2018 | | | |  | | | |  | | |
|  | | | Sant'Anna 2014 | | | |  | | | |  | | |
|  | | | Steinhoff 2017 | | | |  | | | |  | | |
|  | | | Xiao 2017 (BMMNC) | | | |  | | | |  | | |
|  | | |  | | | |  | | | |  | | |
| **Fig 12C** | | | | | | | | | | | | | |
| ***< 1 mio*** | | | ***1 to ≤ 10 mio*** | | | | ***> 10 to ≤ 100 mio*** | | | | ***> 100 mio*** | | |
| – | | | Nasseri 2014 | | | | Patila 2014 | | | | Bartunek 2013 | | |
|  | | | Pokushalov 2010 | | | | Perin 2015 (25 mio) | | | | Perin 2015 (150 mio) | | |
|  | | | Santoso 2014 | | | | Perin 2015 (75 mio) | | | | Vrtovec 2011 | | |
|  | | | Seth 2006 | | | |  | | | |  | | |
|  | | | Steinhoff 2017 | | | |  | | | |  | | |
|  | | | Trifunovic 2015 | | | |  | | | |  | | |
|  | | |  | | | |  | | | |  | | |
| **Fig 12D** | | | | | | | | | | | | | |
| ***< 1 mio*** | | | ***1 to ≤ 10 mio*** | | | | ***> 10 to ≤ 100 mio*** | | | | ***> 100 mio*** | | |
| Ang 2008 (ICI) | | | Assmus 2013 (low dose) | | | | Frljak 2018 | | | | Bartunek 2013 | | |
| Ang 2008 (IMI) | | | Assmus 2013 (high dose) | | | | Mathiasen 2015 | | | | Bartunek 2017 (low dose) | | |
|  | | | Henry 2014 (ischemic) | | | | Maureira 2012 | | | | Bartunek 2017 (mid dose) | | |
|  | | | Henry 2014 (non-ischemic) | | | | Patel 2005 | | | | Bartunek 2017 (high dose) | | |
|  | | | Martino 2015 | | | | Patel 2015 (ischemic) | | | | Perin 2015 (150 mio) | | |
|  | | | Nasseri 2014 | | | | Patel 2015 (non-ischemic) | | | | Vrtovec 2011 | | |
|  | | | Noiseux 2016 | | | | Perin 2015 (25 mio) | | | | Xiao 2017 (BMMSC) | | |
|  | | | Patel 2016 | | | | Perin 2015 (75 mio) | | | |  | | |
|  | | | Perin 2011 | | | | Zhao 2008 | | | |  | | |
|  | | | Perin 2012a | | | |  | | | |  | | |
|  | | | Perin 2012b | | | |  | | | |  | | |
|  | | | Pokushalov 2010 | | | |  | | | |  | | |
|  | | | Sant'Anna 2014 | | | |  | | | |  | | |
|  | | | Santoso 2014 | | | |  | | | |  | | |
|  | | | Seth 2006 | | | |  | | | |  | | |
|  | | | Steinhoff 2017 | | | |  | | | |  | | |
|  | | | Trifunovic 2015 | | | |  | | | |  | | |
|  | | | Wang 2015 | | | |  | | | |  | | |
|  | | | Xiao 2017 (BMMNC) | | | |  | | | |  | | |
|  | | |  | | | |  | | | |  | | |
| **Fig 12E** | | | | | | | | | | | | | |
| ***< 1 mio*** | | | ***1 to ≤ 10 mio*** | | | | ***> 10 to ≤ 100 mio*** | | | | ***> 100 mio*** | | |
| – | | | Choudhury 2017 (ICI) | | | | Patel 2015 (ischemic) | | | | Bartunek 2017 (low dose) | | |
|  | | | Choudhury 2017 (TESI) | | | | Patel 2015 (non-ischemic) | | | | Bartunek 2017 (mid dose) | | |
|  | | | Heldman 2014 (BMMNC) | | | | Patila 2014 | | | | Bartunek 2017 (high dose) | | |
|  | | | Henry 2014 (ischemic) | | | | Perin 2015 (25 mio) | | | | Heldman 2014 (BMMSC) | | |
|  | | | Henry 2014 (non-ischemic) | | | | Perin 2015 (75 mio) | | | | Perin 2015 (150 mio) | | |
|  | | | Hu 2011 | | | |  | | | | Vrtovec 2011 | | |
|  | | | Martino 2015 | | | |  | | | | Xiao 2017 (BMMSC) | | |
|  | | | Patel 2016 | | | |  | | | |  | | |
|  | | | Pokushalov 2010 | | | |  | | | |  | | |
|  | | | Qi 2018 | | | |  | | | |  | | |
|  | | | Sant'Anna 2014 | | | |  | | | |  | | |
|  | | | Trifunovic 2015 | | | |  | | | |  | | |
|  | | | Xiao 2017 (BMMNC) | | | |  | | | |  | | |
|  | | |  | | | |  | | | |  | | |
| **Fig 12F** | | | | | | | | | | | | | |
| ***<1 Mio*** | | | ***1-10 Mio*** | | | | ***10-100 Mio*** | | | | ***>100 Mio*** | | |
| Ang 2008 (ICI) | | | Assmus 2013 (low dose) | | | | Frljak 2018 | | | | Bartunek 2013 | | |
| Ang 2008 (IMI) | | | Assmus 2013 (high dose) | | | | Mathiasen 2015 | | | | Bartunek 2017 (low dose) | | |
|  | | | Henry 2014 (ischemic) | | | | Maureira 2012 | | | | Bartunek 2017 (mid dose) | | |
|  | | | Henry 2014 (non-ischemic) | | | | Patel 2005 | | | | Bartunek 2017 (high dose) | | |
|  | | | Martino 2015 | | | | Perin 2015 (25 mio) | | | | Perin 2015 (150 mio) | | |
|  | | | Nasseri 2014 | | | | Perin 2015 (75 mio) | | | | Vrtovec 2011 | | |
|  | | | Noiseux 2016 | | | |  | | | |  | | |
|  | | | Patel 2016 | | | |  | | | |  | | |
|  | | | Perin 2011 | | | |  | | | |  | | |
|  | | | Perin 2012a | | | |  | | | |  | | |
|  | | | Perin 2012b | | | |  | | | |  | | |
|  | | | Pokushalov 2010 | | | |  | | | |  | | |
|  | | | Sant'Anna 2014 | | | |  | | | |  | | |
|  | | | Santoso 2014 | | | |  | | | |  | | |
|  | | | Steinhoff 2017 | | | |  | | | |  | | |
|  | | |  | | | |  | | | |  | | |
| **Fig 12G** | | | | | | | | | | | | | |
| ***< 1 mio*** | | | ***1 to ≤ 10 mio*** | | | | ***> 10 to ≤ 100 mio*** | | | | ***> 100 mio*** | | |
| – | | | Choudhury 2017 (ICI) | | | | Perin 2015 (25 mio) | | | | Bartunek 2017 (low dose) | | |
|  | | | Choudhury 2017 (TESI) | | | | Perin 2015 (75 mio) | | | | Bartunek 2017 (mid dose) | | |
|  | | | Heldman 2014 (BMMNC) | | | |  | | | | Bartunek 2017 (high dose) | | |
|  | | | Henry 2014 (ischemic) | | | |  | | | | Heldman 2014 (BMMSC) | | |
|  | | | Henry 2014 (non-ischemic) | | | |  | | | | Perin 2015 (150 mio) | | |
|  | | | Hu 2011 | | | |  | | | | Vrtovec 2011 | | |
|  | | | Martino 2015 | | | |  | | | |  | | |
|  | | | Patel 2016 | | | |  | | | |  | | |
|  | | | Pokushalov 2010 | | | |  | | | |  | | |
|  | | | Sant'Anna 2014 | | | |  | | | |  | | |
|  | | |  | | | |  | | | |  | | |
| **Fig 12H** | | | | | | | | | | | | | |
| ***< 1 mio*** | | | ***1 to ≤ 10 mio*** | | | | ***> 10 to ≤ 100 mio*** | | | | ***> 100 mio*** | | |
| – | | | Assmus 2013 (low dose) | | | | Mathiasen 2015 | | | | Xiao 2017 (BMMSC) | | |
|  | | | Assmus 2013 (high dose) | | | | Patel 2005 | | | |  | | |
|  | | | Choudhury 2017 (ICI) | | | | Zhao 2008 | | | |  | | |
|  | | | Choudhury 2017 (TESI) | | | |  | | | |  | | |
|  | | | Nasseri 2014 | | | |  | | | |  | | |
|  | | | Perin 2011 | | | |  | | | |  | | |
|  | | | Perin 2012a | | | |  | | | |  | | |
|  | | | Pokushalov 2010 | | | |  | | | |  | | |
|  | | | Sant'Anna 2014 | | | |  | | | |  | | |
|  | | | Trifunovic 2015 | | | |  | | | |  | | |
|  | | | Xiao 2017 BMMNC | | | |  | | | |  | | |
|  | | |  | | | |  | | | |  | | |
| **Fig 12I** | | | | | | | | | | | | | |
| ***< 1 mio*** | | | ***1 to ≤ 10 mio*** | | | | ***> 10 to ≤ 100 mio*** | | | | ***> 100 mio*** | | |
| – | | | Choudhury 2017 (ICI) | | | | – | | | | Xiao 2017 (BMMSC) | | |
|  | | | Choudhury 2017 (TESI) | | | |  | | | |  | | |
|  | | | Pokushalov 2010 | | | |  | | | |  | | |
|  | | | Sant'Anna 2014 | | | |  | | | |  | | |
|  | | | Seth 2006 | | | |  | | | |  | | |
|  | | | Trifunovic 2015 | | | |  | | | |  | | |
|  | | | Xiao 2017 (BMMNC) | | | |  | | | |  | | |
