# Supplementary material for: Impact of procedural variability and study design quality on the efficacy of cell-based therapies for heart failure - a meta-analysis
Source: PLoS One. 2022 Jan 5;17(1):e0261462. doi: 10.1371/journal.pone.0261462 (PMC8730409; doi:10.1371/journal.pone.0261462)
Supplement: S4 Table — The first number represents the number of comparisons, the second number indicates the number of participants, the third number represents the effect size [95% confidence interval] and the fourth number indicates the value of I2 index. (DOCX) [file pone.0261462.s006.docx]

| **Group** | **Subgroup** | **Follow-up**  **(months)** | **LVEF change**  **(%)** | **LV reverse remodeling**  **(change from baseline)** | **BNP/NT-proBNP levels**  **(change from baseline)** | **Exercise capacity**  **(change from baseline)** | **NYHA**  **(mean at endpoint)** | **Quality of life**  **(mean at endpoint)** |
| --- | --- | --- | --- | --- | --- | --- | --- | --- |
| Overall | – | ≤ 6 | 44 | 33 | 4 | 16 | 19 | 10 |
|  |  |  | 1904 | 1469 | 288 | 745 | 655 | 528 |
|  |  |  | 2.72 [1.52, 3.92] | -0.10 [-0.32, 0.11] | -1.29 [-2.40, -0.17] | 35.42 [12.31, 58.53] | -0.42 [-0.82, -0.03] | -0.52 [-1.13, 0.10] |
|  |  | > 6 to ≤ 12 | 27 | 21 | 1 | 7 | 11 | 7 |
|  |  |  | 1046 | 866 | 35 | 265 | 392 | 305 |
|  |  |  | 2.40 [0.75, 4.05] | -0.35 [-0.59, -0.11] | -5.83 [-7.47, -4.20] | 47.73 [-20.00, 115.46] | -0.50 [-0.96, -0.03] | -0.62 [-1.62, 0.37] |
| HF type | Ischemic HF | ≤ 6 | 25 | 21 | 1 | 7 | 12 | 4 |
|  |  |  | 1121 | 970 | 50 | 294 | 477 | 182 |
|  |  |  | 2.49 [0.80, 4.18] | -0.10 [-0.35, 0.14] | -0.16 [-0.72, 0.41] | 30.89 [-1.52, 63.30] | -0.41 [-0.92, 0.09] | -1.12 [-2.41, 0.17] |
|  |  | > 6 to ≤ 12 | 15 | 11 | 0 | 3 | 4 | 3 |
|  |  |  | 696 | 568 | 0 | 79 | 168 | 138 |
|  |  |  | 2.06 [-0.31, 4.43] | -0.32 [-0.49, -0.15] | Not estimable | 65.66 [-38.85, 170.18] | -0.52 [-1.34, 0.29] | -1.23 [-3.37, 0.90] |
|  | Non-ischemic HF | ≤ 6 | 9 | 5 | 2 | 5 | 3 | 2 |
|  |  |  | 371 | 258 | 108 | 256 | 73 | 121 |
|  |  |  | 3.79 [1.88, 5.70] | 0.01 [-0.89, 0.91] | -2.34 [-2.84, -1.84] | 35.54 [-15.12, 86.20] | -0.46 [-0.94, 0.02] | 0.36 [-0.00, 0.72] |
|  |  | > 6 to ≤ 12 | 7 | 4 | 1 | 5 | 4 | 2 |
|  |  |  | 257 | 185 | 35 | 209 | 149 | 114 |
|  |  |  | 3.00 [0.02, 5.98] | -0.99 [-2.10, 0.13] | -5.83 [-7.47, -4.20] | 32.49 [-74.59, 139.58] | -0.53 [-0.84, -0.21] | 0.20 [-0.27, 0.68] |
|  | Both | ≤ 6 | 3 | 3 | 0 | 0 | 0 | 0 |
|  |  |  | 57 | 55 | 0 | 0 | 0 | 0 |
|  |  |  | 2.64 [-1.33, 6.62] | -0.35 [-1.14, 0.44] | Not estimable | Not estimable | Not estimable | Not estimable |
|  |  | > 6 to ≤ 12 | 3 | 3 | 0 | 0 | 0 | 0 |
|  |  |  | 56 | 54 | 0 | 0 | 0 | 0 |
|  |  |  | 1.70 [-3.56, 6.96] | 0.21 [-0.66, 1.08] | Not estimable | Not estimable | Not estimable | Not estimable |
| Cell source | Autologous | ≤ 6 | 34 | 26 | 3 | 12 | 15 | 6 |
|  |  |  | 1492 | 1228 | 158 | 550 | 550 | 303 |
|  |  |  | 2.74 [1.35, 4.12] | -0.11 [-0.36, 0.14] | -1.62 [-3.16, -0.08] | 34.08 [9.09, 59.07] | -0.43 [-0.89, 0.02] | -0.62 [-1.72, 0.47] |
|  |  | > 6 to ≤ 12 | 22 | 15 | 1 | 7 | 8 | 5 |
|  |  |  | 953 | 753 | 35 | 265 | 317 | 252 |
|  |  |  | 2.34 [0.53, 4.15] | -0.46 [-0.73, -0.19] | -5.83 [-7.47, -4.20] | 47.73 [-20.00, 115.46] | -0.53 [-1.09, 0.04] | -0.70 [-2.05, 0.64] |
|  | Allogeneic | ≤ 6 | 3 | 3 | 0 | 0 | 0 | 0 |
|  |  |  | 57 | 55 | 0 | 0 | 0 | 0 |
|  |  |  | 2.64 [-1.33, 6.62] | -0.35 [-1.14, 0.44] | Not estimable | Not estimable | Not estimable | Not estimable |
|  |  | > 6 to ≤ 12 | 3 | 3 | 0 | 0 | 0 | 0 |
|  |  |  | 56 | 54 | 0 | 0 | 0 | 0 |
|  |  |  | 1.70 [-3.56, 6.96] | 0.21 [-0.66, 1.08] | Not estimable | Not estimable | Not estimable | Not estimable |
| Cell origin | Myoblasts | ≤ 6 | 3 | 1 | 0 | 3 | 2 | 2 |
|  |  |  | 127 | 7 | 0 | 63 | 52 | 63 |
|  |  |  | -0.01 [-2.10, 2.09] | 0.34 [-1.18, 1.86] | Not estimable | 89.88 [34.66, 145.10] | -0.79 [-1.57, -0.00] | -0.49 [-1.15, 0.17] |
|  |  | > 6 to ≤ 12 | 1 | 2 | 0 | 0 | 1 | 1 |
|  |  |  | 9 | 31 | 0 | 0 | 23 | 25 |
|  |  |  | -1.25 [-14.40, 11.90] | -0.17 [-0.90, 0.56] | Not estimable | Not estimable | -1.10 [-1.51, -0.69] | -1.04 [-1.89, -0.19] |
|  | BM-derived cells | ≤ 6 | 37 | 29 | 3 | 12 | 15 | 6 |
|  |  |  | 1549 | 1283 | 158 | 550 | 550 | 303 |
|  |  |  | 2.74 [1.41, 4.07] | -0.12 [-0.36, 0.12] | -1.62 [-3.16, -0.08] | 34.08 [9.09, 59.07] | -0.43 [-0.89, 0.02] | -0.62 [-1.72, 0.47] |
|  |  | > 6 to ≤ 12 | 25 | 18 | 1 | 7 | 8 | 5 |
|  |  |  | 1009 | 807 | 35 | 265 | 317 | 252 |
|  |  |  | 2.30 [0.57, 4.03] | -0.39 [-0.65, -0.13] | -5.83 [-7.47, -4.20] | 47.73 [-20.00, 115.46] | -0.53 [-1.09, 0.04] | -0.70 [-2.05, 0.64] |
|  | AT-derived cells | ≤ 6 | 1 | 1 | 0 | 0 | 1 | 0 |
|  |  |  | 27 | 27 | 0 | 0 | 25 | 0 |
|  |  |  | 0.73 [-1.04, 2.50] | 0.25 [-0.52, 1.01] | Not estimable | Not estimable | -0.04 [-0.58, 0.50] | Not estimable |
|  |  | > 6 to ≤ 12 | 0 | 0 | 0 | 0 | 1 | 0 |
|  |  |  | 0 | 0 | 0 | 0 | 24 | 0 |
|  |  |  | Not estimable | Not estimable | Not estimable | Not estimable | -0.11 [-0.69, 0.47] | Not estimable |
|  | Perinatal cells | ≤ 6 | 2 | 1 | 0 | 0 | 1 | 1 |
|  |  |  | 78 | 28 | 0 | 0 | 28 | 28 |
|  |  |  | 6.46 [2.78, 10.14] | 0.32 [-0.43, 1.06] | Not estimable | Not estimable | 0.07 [-0.35, 0.49] | 0.01 [-0.73, 0.75] |
|  |  | > 6 to ≤ 12 | 1 | 1 | 0 | 0 | 1 | 1 |
|  |  |  | 28 | 28 | 0 | 0 | 28 | 28 |
|  |  |  | 5.22 [0.84, 9.60] | 0.03 [-0.71, 0.77] | Not estimable | Not estimable | -0.03 [-0.50, 0.44] | 0.17 [-0.57, 0.91] |
|  | Cardiac-derived cells | ≤ 6 | 1 | 1 | 1 | 0 | 0 | 1 |
|  |  |  | 123 | 124 | 130 | 0 | 0 | 134 |
|  |  |  | 0.90 [-3.29, 5.09] | -0.41 [-0.79, -0.03] | -0.37 [-0.74, -0.00] | Not estimable | Not estimable | -0.36 [-0.72, 0.00] |
|  |  | > 6 to ≤ 12 | 0 | 0 | 0 | 0 | 0 | 0 |
|  |  |  | 0 | 0 | 0 | 0 | 0 | 0 |
|  |  |  | Not estimable | Not estimable | Not estimable | Not estimable | Not estimable | Not estimable |
| Cell type | BMMNCs | ≤ 6 | 19 | 12 | 3 | 6 | 10 | 6 |
|  |  |  | 801 | 538 | 110 | 341 | 377 | 303 |
|  |  |  | 3.56 [2.24, 4.87] | 0.02 [-0.36, 0.39] | -1.17 [-3.18, 0.83] | 11.11 [-26.85, 49.06] | -0.44 [-0.95, 0.07] | -0.62 [-1.72, 0.47] |
|  |  | > 6 to ≤ 12 | 13 | 7 | 0 | 3 | 7 | 5 |
|  |  |  | 499 | 324 | 0 | 139 | 293 | 252 |
|  |  |  | 2.55 [0.09, 5.02] | -0.29 [-0.52, -0.06] | -0.48 [-1.26, 0.31] | 14.16 [-28.45, 56.78] | -0.50 [-1.10, 0.10] | -0.70 [-2.05, 0.64] |
|  | CD34+ cells | ≤ 6 | 2 | 2 | 1 | 1 | 1 | 0 |
|  |  |  | 68 | 68 | 48 | 48 | 20 | 0 |
|  |  |  | 8.23 [4.72, 11.75] | -1.50 [-3.22, 0.22] | -2.54 [-3.32, -1.76] | 116.77 [97.50, 136.04] | -2.00 [-2.66, -1.34] | Not estimable |
|  |  | > 6 to ≤ 12 | 1 | 1 | 1 | 1 | 0 | 0 |
|  |  |  | 45 | 44 | 35 | 45 | 0 | 0 |
|  |  |  | 6.51 [5.14, 7.88] | -1.82 [-2.54, -1.10] | -5.83 [-7.47, -4.20] | 132.34 [104.66, 160.02] | Not estimable | Not estimable |
|  | CD133+ cells | ≤ 6 | 3 | 3 | 1 | 1 | 1 | 0 |
|  |  |  | 107 | 136 | 54 | 42 | 53 | 0 |
|  |  |  | -2.06 [-5.79, 1.68] | 0.18 [-0.16, 0.52] | 0.74 [0.19, 1.30] | 6.94 [-46.63, 60.51] | 0.54 [0.17, 0.91] | Not estimable |
|  |  | > 6 to ≤ 12 | 0 | 0 | 0 | 0 | 0 | 0 |
|  |  |  | 0 | 0 | 0 | 0 | 0 | 0 |
|  |  |  | Not estimable | Not estimable | Not estimable | Not estimable | Not estimable | Not estimable |
|  | ALDH+ cells | ≤ 6 | 1 | 1 | 0 | 0 | 1 | 0 |
|  |  |  | 20 | 20 | 0 | 0 | 20 | 0 |
|  |  |  | -2.00 [-9.12, 5.12] | -0.29 [-1.17, 0.59] | Not estimable | Not estimable | 0.20 [-0.16, 0.56] | Not estimable |
|  |  | > 6 to ≤ 12 | 0 | 0 | 0 | 0 | 0 | 0 |
|  |  |  | 0 | 0 | 0 | 0 | 0 | 0 |
|  |  |  | Not estimable | Not estimable | Not estimable | Not estimable | Not estimable | Not estimable |
|  | BMMSCs | ≤ 6 | 2 | 1 | 0 | 1 | 2 | 0 |
|  |  |  | 85 | 55 | 0 | 27 | 80 | 0 |
|  |  |  | 6.10 [4.31, 7.89] | 0.32 [-0.24, 0.89] | Not estimable | 6.60 [-25.17, 38.37] | -0.41 [-0.95, 0.12] | Not estimable |
|  |  | > 6 to ≤ 12 | 2 | 1 | 0 | 1 | 1 | 0 |
|  |  |  | 48 | 24 | 0 | 25 | 24 | 0 |
|  |  |  | 2.43 [-7.75, 12.60] | -0.55 [-1.42, 0.31] | Not estimable | 26.30 [-35.17, 87.77] | -0.80 [-2.16, 0.56] | Not estimable |
|  | Cardiopoietic cells | ≤ 6 | 4 | 4 | 0 | 1 | 0 | 0 |
|  |  |  | 263 | 263 | 0 | 36 | 0 | 0 |
|  |  |  | 0.98 [-1.95, 3.91] | -0.19 [-0.47, 0.10] | Not estimable | 77.00 [64.28, 89.72] | Not estimable | Not estimable |
|  |  | > 6 to ≤ 12 | 3 | 3 | 0 | 0 | 0 | 0 |
|  |  |  | 220 | 220 | 0 | 0 | 0 | 0 |
|  |  |  | -0.12 [-2.95, 2.71] | -0.39 [-0.66, -0.12] | Not estimable | Not estimable | Not estimable | Not estimable |
|  | STRO-3^+^ MPCs | ≤ 6 | 3 | 3 | 0 | 0 | 0 | 0 |
|  |  |  | 57 | 55 | 0 | 0 | 0 | 0 |
|  |  |  | 2.64 [-1.33, 6.62] | -0.35 [-1.14, 0.44] | Not estimable | Not estimable | Not estimable | Not estimable |
|  |  | > 6 to ≤ 12 | 3 | 3 | 3 | 0 | 0 | 0 |
|  |  |  | 56 | 54 | 52 | 0 | 0 | 0 |
|  |  |  | 1.70 [-3.56, 6.96] | 0.21 [-0.66, 1.08] | -0.04 [-0.69, 0.61] | Not estimable | Not estimable | Not estimable |
|  | Lxmyelocel-T | ≤ 6 | 3 | 3 | 0 | 2 | 0 | 0 |
|  |  |  | 148 | 148 | 0 | 56 | 0 | 0 |
|  |  |  | 1.07 [-1.16, 3.29] | -0.22 [-2.01, 1.57] | Not estimable | 32.54 [-41.34, 106.42] | Not estimable | Not estimable |
|  |  | > 6 to ≤ 12 | 3 | 3 | 0 | 2 | 0 | 0 |
|  |  |  | 141 | 141 | 0 | 56 | 0 | 0 |
|  |  |  | 0.96 [-1.88, 3.79] | -0.53 [-1.74, 0.67] | Not estimable | 58.62 [-124.99, 242.22] | Not estimable | Not estimable |
| Cell processing | Primary cells | ≤ 6 | 26 | 19 | 3 | 8 | 14 | 6 |
|  |  |  | 1023 | 789 | 158 | 431 | 495 | 303 |
|  |  |  | 2.88 [1.22, 4.53] | -0.09 [-0.38, 0.21] | -1.62 [-3.16, -0.08] | 28.35 [-12.28, 68.99] | -0.40 [-0.87, 0.08] | -0.62 [-1.72, 0.47] |
|  |  | > 6 to ≤ 12 | 14 | 8 | 1 | 4 | 8 | 5 |
|  |  |  | 544 | 368 | 35 | 184 | 317 | 252 |
|  |  |  | 3.11 [1.04, 5.18] | -0.49 [-0.89, -0.09] | -5.83 [-7.47, -4.20] | 47.95 [-34.38, 130.29] | -0.45 [-1.02, 0.11] | -0.70 [-2.05, 0.64] |
|  | In vitro cultured cells | ≤ 6 | 18 | 14 | 1 | 7 | 5 | 3 |
|  |  |  | 881 | 680 | 130 | 182 | 160 | 91 |
|  |  |  | 2.45 [0.72, 4.18] | -0.13 [-0.46, 0.21] | -0.37 [-0.74, -0.00] | 49.72 [15.80, 83.64] | -0.47 [-0.91, -0.03] | -0.32 [-0.81, 0.17] |
|  |  | > 6 to ≤ 12 | 13 | 13 | 0 | 3 | 3 | 2 |
|  |  |  | 502 | 498 | 0 | 81 | 75 | 53 |
|  |  |  | 1.32 [-0.47, 3.11] | -0.25 [-0.56, 0.05] | Not estimable | 48.30 [-87.04, 183.64] | -0.62 [-1.46, 0.22] | -0.42 [-1.61, 0.77] |
| Surgical intervention | Revascularization | ≤ 6 | 10 | 7 | 0 | 2 | 4 | 0 |
|  |  |  | 320 | 195 | 0 | 99 | 137 | 0 |
|  |  |  | 3.68 [-0.32, 7.69] | -0.25 [-0.88, 0.39] | Not estimable | 21.79 [-7.28, 50.87] | -0.60 [-1.48, 0.28] | Not estimable |
|  |  | > 6 to ≤ 12 | 3 | 1 | 0 | 1 | 1 | 0 |
|  |  |  | 115 | 50 | 0 | 30 | 30 | 0 |
|  |  |  | 5.65 [-3.05, 14.35] | -0.63 [-1.20, -0.06] | Not estimable | 161.00 [93.42, 228.58] | -0.26 [-0.54, 0.02] | Not estimable |
|  | No revascularization | ≤ 6 | 27 | 22 | 3 | 10 | 11 | 6 |
|  |  |  | 1229 | 1088 | 158 | 451 | 413 | 303 |
|  |  |  | 2.59 [1.44, 3.73] | -0.10 [-0.37, 0.16] | -1.62 [-3.16, -0.08] | 37.13 [9.16, 65.10] | -0.37 [-0.90, 0.16] | -0.62 [-1.72, 0.47] |
|  |  | > 6 to ≤ 12 | 22 | 17 | 1 | 7 | 7 | 5 |
|  |  |  | 894 | 757 | 35 | 331 | 287 | 252 |
|  |  |  | 1.80 [0.09, 3.51] | -0.37 [-0.65, -0.10] | -5.83 [-7.47, -4.20] | 62.51 [-11.37, 136.40] | -0.57 [-1.16, 0.02] | -0.70 [-2.05, 0.64] |
| Cell delivery route | CABG+IMI | ≤ 6 | 9 | 6 | 0 | 1 | 4 | 0 |
|  |  |  | 309 | 184 | 0 | 42 | 137 | 0 |
|  |  |  | 4.18 [-0.11, 8.47] | -0.23 [-0.93, 0.47] | Not estimable | 6.94 [-46.63, 60.51] | -0.60 [-1.48, 0.28] | Not estimable |
|  |  | > 6 to ≤ 12 | 2 | 0 | 0 | 1 | 1 | 0 |
|  |  |  | 65 | 0 | 0 | 30 | 30 | 0 |
|  |  |  | 5.71 [-7.86, 19.27] | Not estimable | Not estimable | 161.00 [93.42, 228.58] | -0.26 [-0.54, 0.02] | Not estimable |
|  | CABG+ICI | ≤ 6 | 1 | 1 | 0 | 1 | 0 | 0 |
|  |  |  | 11 | 11 | 0 | 57 | 0 | 0 |
|  |  |  | -1.20 [-6.75, 4.35] | -0.46 [-1.81, 0.89] | Not estimable | 28.00 [-6.62, 62.62] | Not estimable | Not estimable |
|  |  | > 6 to ≤ 12 | 1 | 1 | 0 | 0 | 0 | 0 |
|  |  |  | 50 | 50 | 0 | 0 | 0 | 0 |
|  |  |  | 5.50 [0.24, 10.76] | -0.63 [-1.20, -0.06] | Not estimable | Not estimable | Not estimable | Not estimable |
|  | TESI | ≤ 6 | 15 | 15 | 2 | 5 | 5 | 3 |
|  |  |  | 791 | 784 | 110 | 228 | 233 | 154 |
|  |  |  | 2.37 [0.77, 3.98] | -0.13 [-0.39, 0.13] | -1.17 [-3.18, 0.83] | 31.02 [-1.72, 63.75] | -0.42 [-1.20, 0.36] | -1.39 [-2.94, 0.15] |
|  |  | > 6 to ≤ 12 | 11 | 11 | 0 | 5 | 2 | 2 |
|  |  |  | 519 | 517 | 0 | 219 | 109 | 109 |
|  |  |  | 1.15 [-1.69, 4.00] | -0.28 [-0.49, -0.08] | Not estimable | 17.77 [-23.77, 59.32] | -0.73 [-2.09, 0.63] | -1.85 [-4.77, 1.08] |
|  | IMI | ≤ 6 | 1 | 1 | 0 | 1 | 1 | 1 |
|  |  |  | 24 | 24 | 0 | 22 | 24 | 24 |
|  |  |  | 3.44 [-0.61, 7.49] | 0.48 [-0.36, 1.32] | Not estimable | 3.60 [-205.06, 212.26] | -0.07 [-0.71, 0.57] | 0.35 [-0.49, 1.18] |
|  |  | > 6 to ≤ 12 | 1 | 1 | 0 | 1 | 1 | 1 |
|  |  |  | 24 | 24 | 0 | 24 | 23 | 24 |
|  |  |  | 0.85 [-3.27, 4.97] | -0.38 [-1.21, 0.46] | Not estimable | 40.41 [-52.59, 133.41] | -0.30 [-1.07, 0.47] | -0.19 [-1.02, 0.64] |
|  | ICI | ≤ 6 | 9 | 5 | 1 | 5 | 5 | 2 |
|  |  |  | 358 | 254 | 48 | 230 | 156 | 125 |
|  |  |  | 3.10 [1.19, 5.02] | -0.10 [-0.53, 0.32] | -2.54 [-3.32, -1.76] | 42.50 [-18.29, 103.28] | -0.26 [-0.49, -0.02] | 0.11 [-0.53, 0.74] |
|  |  | > 6 to ≤ 12 | 8 | 3 | 1 | 1 | 4 | 2 |
|  |  |  | 295 | 160 | 35 | 88 | 155 | 119 |
|  |  |  | 2.50 [-0.18, 5.18] | -0.57 [-1.73, 0.59] | -5.83 [-7.47, -4.20] | -58.70 [-195.25, 77.85] | -0.49 [-0.77, -0.22] | 0.27 [-0.10, 0.63] |
| Delivered cell dose | < 1 mio | ≤ 6 | 2 | 2 | 0 | 0 | 0 | 0 |
|  |  |  | 25 | 25 | 0 | 0 | 0 | 0 |
|  |  |  | 1.39 [-3.30, 6.08] | -0.77 [-1.68, 0.14] | Not estimable | Not estimable | Not estimable | Not estimable |
|  |  | > 6 to ≤ 12 | 0 | 0 | 0 | 0 | 0 | 0 |
|  |  |  | 0 | 0 | 0 | 0 | 0 | 0 |
|  |  |  | Not estimable | Not estimable | Not estimable | Not estimable | Not estimable | Not estimable |
|  | > 1 to ≤ 10 mio | ≤ 6 | 19 | 15 | 1 | 8 | 11 | 6 |
|  |  |  | 896 | 743 | 50 | 379 | 416 | 303 |
|  |  |  | 2.00 [0.17, 3.83] | 0.14 [-0.14, 0.41] | -0.16 [-0.72, 0.41] | 11.08 [-25.37, 47.54] | -0.26 [-0.82, 0.30] | -0.62 [-1.72, 0.47] |
|  |  | > 6 to ≤ 12 | 13 | 10 | 0 | 5 | 7 | 5 |
|  |  |  | 558 | 465 | 0 | 195 | 293 | 252 |
|  |  |  | 3.28 [0.97, 5.59] | -0.34 [-0.66, -0.02] | Not estimable | 32.21 [-68.06, 132.49] | -0.50 [-1.10, 0.10] | -0.70 [-2.05, 0.64] |
|  | > 10 to ≤ 100 mio | ≤ 6 | 9 | 6 | 1 | 1 | 3 | 0 |
|  |  |  | 272 | 185 | 60 | 60 | 109 | 0 |
|  |  |  | 5.23 [2.74, 7.73] | -0.42 [-1.38, 0.53] | -2.20 [-2.85, -1.55] | 45.00 [35.10, 54.90] | -0.96 [-1.79, -0.13] | Not estimable |
|  |  | > 6 to ≤ 12 | 5 | 2 | 0 | 0 | 0 | 0 |
|  |  |  | 120 | 35 | 0 | 0 | 0 | 0 |
|  |  |  | -0.44 [-3.22, 2.33] | 0.61 [-0.20, 1.42] | Not estimable | Not estimable | Not estimable | Not estimable |
|  | > 100 mio | ≤ 6 | 7 | 6 | 1 | 3 | 1 | 0 |
|  |  |  | 356 | 330 | 48 | 111 | 25 | 0 |
|  |  |  | 2.54 [-0.16, 5.23] | -0.33 [-0.63, -0.03] | -2.54 [-3.32, -1.76] | 68.68 [20.96, 116.40] | -0.80 [-1.60, -0.00] | Not estimable |
|  |  | > 6 to ≤ 12 | 7 | 6 | 2 | 2 | 1 | 0 |
|  |  |  | 331 | 307 | 51 | 70 | 24 | 0 |
|  |  |  | 1.70 [-2.11, 5.50] | -0.65 [-1.08, -0.22] | -2.76 [-8.73, 3.22] | 83.02 [-20.65, 186.68] | -0.80 [-2.16, 0.56] | Not estimable |
| selection bias | high/unclear risk | ≤ 6 | 25 | − | − | − | − | − |
|  |  |  | 887 | − | − | − | − | − |
|  |  |  | 4.00 [2.71, 5.28] | − | − | − | − | − |
|  |  | > 6 to ≤ 12 | 17 | − | − | − | − | − |
|  |  |  | 525 | − | − | − | − | − |
|  |  |  | 3.38 [1.40, 5.36] | − | − | − | − | − |
|  | low risk | ≤ 6 | 19 | − | − | − | − | − |
|  |  |  | 1017 | − | − | − | − | − |
|  |  |  | 1.53 [-0.35, 3.41] | − | − | − | − | − |
|  |  | > 6 to ≤ 12 | 10 | − | − | − | − | − |
|  |  |  | 521 | − | − | − | − | − |
|  |  |  | 1.04 [-1.00, 3.09] | − | − | − | − | − |
| performance bias | high/unclear risk | ≤ 6 | 18 | − | − | − | − | − |
|  |  |  | 642 | − | − | − | − | − |
|  |  |  | 5.31 [3.97, 6.64] | − | − | − | − | − |
|  |  | > 6 to ≤ 12 | 10 | − | − | − | − | − |
|  |  |  | 329 | − | − | − | − | − |
|  |  |  | 4.82 [2.64, 7.01] | − | − | − | − | − |
|  | low risk | ≤ 6 | 26 | − | − | − | − | − |
|  |  |  | 1262 | − | − | − | − | − |
|  |  |  | 1.19 [-0.12, 2.51] | − | − | − | − | − |
|  |  | > 6 to ≤ 12 | 17 | − | − | − | − | − |
|  |  |  | 717 | − | − | − | − | − |
|  |  |  | 1.06 [-0.41, 2.53] | − | − | − | − | − |
| detection bias | high/unclear risk | ≤ 6 | 8 | − | − | − | − | − |
|  |  |  | 258 | − | − | − | − | − |
|  |  |  | 3.99 [1.49, 6.50] | − | − | − | − | − |
|  |  | > 6 to ≤ 12 | 3 | − | − | − | − | − |
|  |  |  | 77 | − | − | − | − | − |
|  |  |  | 3.43 [-7.36, 14.21] | − | − | − | − | − |
|  | low risk | ≤ 6 | 36 | − | − | − | − | − |
|  |  |  | 1646 | − | − | − | − | − |
|  |  |  | 2.45 [1.10, 3.80] | − | − | − | − | − |
|  |  | > 6 to ≤ 12 | 24 | − | − | − | − | − |
|  |  |  | 969 | − | − | − | − | − |
|  |  |  | 2.17 [0.57, 3.76] | − | − | − | − | − |
