# Supplementary material for: Impact of procedural variability and study design quality on the efficacy of cell-based therapies for heart failure - a meta-analysis
Source: PLoS One. 2022 Jan 5;17(1):e0261462. doi: 10.1371/journal.pone.0261462 (PMC8730409; doi:10.1371/journal.pone.0261462)
Supplement: S5 Table — The first number represents the number of comparisons, the second number indicates the number of participants, the third number represents the effect size [95% confidence interval] and the fourthl number indicates the value of I2 index. (DOCX) [file pone.0261462.s007.docx]

| **Group** | **Subgroup** | **Follow up (months)** | **Mortality** | | | **MACEs** | | | **SAEs** | | |  |
| --- | --- | --- | --- | --- | --- | --- | --- | --- | --- | --- | --- | --- |
| Overall | – | ≤ 6 | 28 | | | 13 | | | 11 | | |  |
|  |  |  | 1404 | | | 689 | | | 633 | | |  |
|  |  |  | 0.88 [0.59, 1.32] | | | 0.95 [0.69, 1.30] | | | 1.10 [0.84, 1.44] | | |  |
|  |  | > 6 to ≤ 12 | 25 | | | 16 | | | 10 | | |  |
|  |  |  | 1410 | | | 841 | | | 760 | | |  |
|  |  |  | 0.74 [0.47, 1.15] | | | 0.86 [0.67, 1.11] | | | 0.87 [0.66, 1.14] | | |  |
|  |  | > 12 | 14 | | | 5 | | | 1 | | |  |
|  |  |  | 644 | | | 190 | | | 97 | | |  |
|  |  |  | 0.58 [0.41, 0.82] | | | 0.58 [0.29, 1.15] | | | 1.40 [1.08, 1.82] | | |  |
| HF type | Ischemic HF | ≤ 6 | 16 | | | 8 | | | 5 | | |  |
|  |  |  | 826 | | | 398 | | | 319 | | |  |
|  |  |  | 0.62 [0.34, 1.13] | | | 0.75 [0.45, 1.25] | | | 0.90 [0.48, 1.71] | | |  |
|  |  | > 6 to ≤ 12 | 14 | | | 7 | | | 6 | | |  |
|  |  |  | 897 | | | 327 | | | 468 | | |  |
|  |  |  | 0.60 [0.31, 1.14] | | | 0.59 [0.39, 0.88] | | | 0.90 [0.56, 1.45] | | |  |
|  |  | > 12 | 7 | | | 0 | | | 0 | | |  |
|  |  |  | 359 | | | 0 | | | 0 | | |  |
|  |  |  | 0.56 [0.28, 1.11] | | | Not estimable | | | Not estimable | | |  |
|  | Non-ischemic HF | ≤ 6 | 7 | | | 1 | | | 0 | | |  |
|  |  |  | 355 | | | 30 | | | 0 | | |  |
|  |  |  | 1.17 [0.53, 2.57] | | | Not estimable | | | Not estimable | | |  |
|  |  | > 6 to ≤ 12 | 6 | | | 5 | | | 1 | | |  |
|  |  |  | 281 | | | 222 | | | 30 | | |  |
|  |  |  | 0.89 [0.39, 2.01] | | | 1.16 [0.71, 1.89] | | | 1.00 [0.14, 7.39] | | |  |
|  |  | > 12 | 2 | | | 0 | | | 0 | | |  |
|  |  |  | 191 | | | 0 | | | 0 | | |  |
|  |  |  | 0.61 [0.31, 1.19] | | | Not estimable | | | Not estimable | | |  |
|  | Both | ≤ 6 | 0 | | | 0 | | | 0 | | |  |
|  |  |  | 0 | | | 0 | | | 0 | | |  |
|  |  |  | Not estimable | | | Not estimable | | | Not estimable | | |  |
|  |  | > 6 to ≤ 12 | 0 | | | 0 | | | 0 | | |  |
|  |  |  | 0 | | | 0 | | | 0 | | |  |
|  |  |  | Not estimable | | | Not estimable | | | Not estimable | | |  |
|  |  | > 12 | 3 | | | 3 | | | 0 | | |  |
|  |  |  | 60 | | | 60 | | | 0 | | |  |
|  |  |  | 0.33 [0.10, 1.15] | | | 0.46 [0.22, 0.95] | | | Not estimable | | |  |
| Cell source | Autologous | ≤ 6 | 23 | | | 8 | | | 5 | | |  |
|  |  |  | 1181 | | | 398 | | | 319 | | |  |
|  |  |  | 0.88 [0.57, 1.37] | | | 0.75 [0.45, 1.25] | | | 0.90 [0.48, 1.71] | | |  |
|  |  | > 6 to ≤ 12 | 21 | | | 12 | | | 7 | | |  |
|  |  |  | 1216 | | | 549 | | | 498 | | |  |
|  |  |  | 0.73 [0.46, 1.16] | | | 0.78 [0.57, 1.06] | | | 0.89 [0.57, 1.39] | | |  |
|  |  | > 12 | 9 | | | 0 | | | 0 | | |  |
|  |  |  | 550 | | | 0 | | | 0 | | |  |
|  |  |  | 0.59 [0.38, 0.93] | | | Not estimable | | | Not estimable | | |  |
|  | Allogeneic | ≤ 6 | 0 | | | 0 | | | 0 | | |  |
|  |  |  | 0 | | | 0 | | | 0 | | |  |
|  |  |  | Not estimable | | | Not estimable | | | Not estimable | | |  |
|  |  | > 6 to ≤ 12 | 0 | | | 0 | | | 0 | | |  |
|  |  |  | 0 | | | 0 | | | 0 | | |  |
|  |  |  | Not estimable | | | Not estimable | | | Not estimable | | |  |
|  |  | > 12 | 3 | | | 3 | | | 0 | | |  |
|  |  |  | 60 | | | 60 | | | 0 | | |  |
|  |  |  | 0.33 [0.10, 1.15] | | | 0.46 [0.22, 0.95] | | | Not estimable | | |  |
| Cell origin | Myoblasts | ≤ 6 | 4 | | | 4 | | | 5 | | |  |
|  |  |  | 164 | | | 157 | | | 180 | | |  |
|  |  |  | 2.27 [0.60, 8.62] | | | 1.49 [0.86, 2.59] | | | 1.28 [0.88, 1.85] | | |  |
|  |  | > 6 to ≤ 12 | 2 | | | 1 | | | 1 | | |  |
|  |  |  | 30 | | | 97 | | | 97 | | |  |
|  |  |  | Not estimable | | | 1.12 [0.69, 1.81] | | | 1.12 [0.69, 1.81] | | |  |
|  |  | > 12 | 1 | | | 1 | | | 1 | | |  |
|  |  |  | 7 | | | 103 | | | 97 | | |  |
|  |  |  | Not estimable | | | 1.28 [0.97, 1.67] | | | 1.40 [1.08, 1.82] | | |  |
|  | BM-derived cells | ≤ 6 | 23 | | | 8 | | | 5 | | |  |
|  |  |  | 1181 | | | 398 | | | 319 | | |  |
|  |  |  | 0.88 [0.57, 1.37] | | | 0.75 [0.45, 1.25] | | | 0.90 [0.48, 1.71] | | |  |
|  |  | > 6 to ≤ 12 | 20 | | | 12 | | | 7 | | |  |
|  |  |  | 1185 | | | 549 | | | 498 | | |  |
|  |  |  | 0.69 [0.44, 1.10] | | | 0.78 [0.57, 1.06] | | | 0.89 [0.57, 1.39] | | |  |
|  |  | > 12 | 12 | | | 3 | | | 0 | | |  |
|  |  |  | 610 | | | 60 | | | 0 | | |  |
|  |  |  | 0.58 [0.40, 0.84] | | | 0.46 [0.22, 0.95] | | | Not estimable | | |  |
|  | AT-derived cells | ≤ 6 | 0 | | | 0 | | | 0 | | |  |
|  |  |  | 0 | | | 0 | | | 0 | | |  |
|  |  |  | Not estimable | | | Not estimable | | | Not estimable | | |  |
|  |  | > 6 to ≤ 12 | 1 | | | 1 | | | 1 | | |  |
|  |  |  | 31 | | | 31 | | | 31 | | |  |
|  |  |  | 5.83 [0.33, 104.22] | | | 1.65 [0.50, 5.42] | | | 0.82 [0.45, 1.49] | | |  |
|  |  | > 12 | 1 | | | 1 | | | 0 | | |  |
|  |  |  | 27 | | | 27 | | | 0 | | |  |
|  |  |  | 0.43 [0.09, 2.00] | | | 0.29 [0.05, 1.62] | | | Not estimable | | |  |
|  | Perinatal cells | ≤ 6 | 1 | | | 0 | | | 0 | | |  |
|  |  |  | 59 | | | 0 | | | 0 | | |  |
|  |  |  | 0.28 [0.06, 1.22] | | | Not estimable | | | Not estimable | | |  |
|  |  | > 6 to ≤ 12 | 1 | | | 1 | | | 0 | | |  |
|  |  |  | 30 | | | 30 | | | 0 | | |  |
|  |  |  | 1.00 [0.07, 14.55] | | | 0.25 [0.03, 1.98] | | | Not estimable | | |  |
|  |  | > 12 | 0 | | | 0 | | | 0 | | |  |
|  |  |  | 0 | | | 0 | | | 0 | | |  |
|  |  |  | Not estimable | | | Not estimable | | | Not estimable | | |  |
|  | Cardiac-derived cells | ≤ 6 | 0 | | | 1 | | | 1 | | |  |
|  |  |  | 0 | | | 134 | | | 134 | | |  |
|  |  |  | Not estimable | | | 0.65 [0.15, 2.79] | | | 0.81 [0.39, 1.71] | | |  |
|  |  | > 6 to ≤ 12 | 1 | | | 1 | | | 1 | | |  |
|  |  |  | 134 | | | 134 | | | 134 | | |  |
|  |  |  | Not estimable | | | 0.68 [0.23, 2.03] | | | 0.78 [0.46, 1.34] | | |  |
|  |  | > 12 | 0 | | | 0 | | | 0 | | |  |
|  |  |  | 0 | | | 0 | | | 0 | | |  |
|  |  |  | Not estimable | | | Not estimable | | | Not estimable | | |  |
| Cell type | BMMNCs | ≤ 6 | 15 | | | 6 | | | 2 | | |  |
|  |  |  | 877 | | | 288 | | | 153 | | |  |
|  |  |  | 1.04 [0.63, 1.72] | | | 0.71 [0.37, 1.37] | | | 0.38 [0.08, 1.83] | | |  |
|  |  | > 6 to ≤ 12 | 13 | | | 7 | | | 4 | | |  |
|  |  |  | 599 | | | 326 | | | 119 | | |  |
|  |  |  | 0.68 [0.31, 1.50] | | | 1.06 [0.64, 1.77] | | | 0.72 [0.47, 1.10] | | |  |
|  |  | > 12 | 5 | | | 0 | | | 0 | | |  |
|  |  |  | 287 | | | 0 | | | 0 | | |  |
|  |  |  | 0.53 [0.28, 1.01] | | | Not estimable | | | Not estimable | | |  |
|  | CD34+ cells | ≤ 6 | 2 | | | 0 | | | 0 | | |  |
|  |  |  | 75 | | | 0 | | | 0 | | |  |
|  |  |  | 0.16 [0.02, 1.25] | | | Not estimable | | | Not estimable | | |  |
|  |  | > 6 to ≤ 12 | 1 | | | 0 | | | 0 | | |  |
|  |  |  | 55 | | | 0 | | | 0 | | |  |
|  |  |  | 0.24 [0.06, 1.03] | | | Not estimable | | | Not estimable | | |  |
|  |  | > 12 | 1 | | | 0 | | | 0 | | |  |
|  |  |  | 110 | | | 0 | | | 0 | | |  |
|  |  |  | 0.42 [0.20, 0.88] | | | Not estimable | | | Not estimable | | |  |
|  | CD133+ cells | ≤ 6 | 2 | | | 2 | | | 3 | | |  |
|  |  |  | 93 | | | 110 | | | 166 | | |  |
|  |  |  | 0.64 [0.06, 6.87] | | | 1.08 [0.07, 16.67] | | | 1.31 [0.86, 1.99] | | |  |
|  |  | > 6 to ≤ 12 | 1 | | | 0 | | | 0 | | |  |
|  |  |  | 57 | | | 0 | | | 0 | | |  |
|  |  |  | Not estimable | | | Not estimable | | | Not estimable | | |  |
|  |  | > 12 | 2 | | | 0 | | | 0 | | |  |
|  |  |  | 117 | | | 0 | | | 0 | | |  |
|  |  |  | 1.00 [0.40, 2.47] | | | Not estimable | | | Not estimable | | |  |
|  | ALDH+ cells | ≤ 6 | 1 | | | 0 | | | 0 | | |  |
|  |  |  | 20 | | | 0 | | | 0 | | |  |
|  |  |  | Not estimable | | | Not estimable | | | Not estimable | | |  |
|  |  | > 6 to ≤ 12 | 0 | | | 0 | | | 0 | | |  |
|  |  |  | 0 | | | 0 | | | 0 | | |  |
|  |  |  | Not estimable | | | Not estimable | | | Not estimable | | |  |
|  |  | > 12 | 0 | | | 0 | | | 0 | | |  |
|  |  |  | 0 | | | 0 | | | 0 | | |  |
|  |  |  | Not estimable | | | Not estimable | | | Not estimable | | |  |
|  | BMMSCs | ≤ 6 | 2 | | | 0 | | | 0 | | |  |
|  |  |  | 86 | | | 0 | | | 0 | | |  |
|  |  |  | 0.56 [0.13, 2.51] | | | Not estimable | | | Not estimable | | |  |
|  |  | > 6 to ≤ 12 | 2 | | | 2 | | | 1 | | |  |
|  |  |  | 56 | | | 55 | | | 30 | | |  |
|  |  |  | 0.38 [0.05, 2.89] | | | 0.69 [0.26, 1.79] | | | 1.16 [0.36, 3.73] | | |  |
|  |  | > 12 | 0 | | | 0 | | | 0 | | |  |
|  |  |  | 0 | | | 0 | | | 0 | | |  |
|  |  |  | Not estimable | | | Not estimable | | | Not estimable | | |  |
|  | Cardiopoietic cells | ≤ 6 | 0 | | | 0 | | | 0 | | |  |
|  |  |  | 0 | | | 0 | | | 0 | | |  |
|  |  |  | Not estimable | | | Not estimable | | | Not estimable | | |  |
|  |  | > 6 to ≤ 12 | 1 | | | 0 | | | 1 | | |  |
|  |  |  | 271 | | | 0 | | | 240 | | |  |
|  |  |  | 1.12 [0.60, 2.10] | | | Not estimable | | | 5.67 [1.71, 18.83] | | |  |
|  |  | > 12 | 1 | | | 0 | | | 0 | | |  |
|  |  |  | 36 | | | 0 | | | 0 | | |  |
|  |  |  | 0.36 [0.04, 3.59] | | | Not estimable | | | Not estimable | | |  |
|  | STRO-3^+^ MPCs | ≤ 6 | 0 | | | 0 | | | 0 | | |  |
|  |  |  | 0 | | | 0 | | | 0 | | |  |
|  |  |  | Not estimable | | | Not estimable | | | Not estimable | | |  |
|  |  | > 6 to ≤ 12 | 0 | | | 0 | | | 0 | | |  |
|  |  |  | 0 | | | 0 | | | 0 | | |  |
|  |  |  | Not estimable | | | Not estimable | | | Not estimable | | |  |
|  |  | > 12 | 3 | | | 3 | | | 0 | | |  |
|  |  |  | 60 | | | 60 | | | 0 | | |  |
|  |  |  | 0.33 [0.10, 1.15] | | | 0.46 [0.22, 0.95] | | | Not estimable | | |  |
|  | Lxmyelocel-T | ≤ 6 | 1 | | | 0 | | | 0 | | |  |
|  |  |  | 30 | | | 0 | | | 0 | | |  |
|  |  |  | 0.86 [0.09, 8.30] | | | Not estimable | | | Not estimable | | |  |
|  |  | > 6 to ≤ 12 | 3 | | | 3 | | | 1 | | |  |
|  |  |  | 178 | | | 168 | | | 109 | | |  |
|  |  |  | 1.05 [0.37, 2.99] | | | 0.65 [0.28, 1.49] | | | 0.66 [0.50, 0.88] | | |  |
|  |  | > 12 | 0 | | | 0 | | | 0 | | |  |
|  |  |  | 0 | | | 0 | | | 0 | | |  |
|  |  |  | Not estimable | | | Not estimable | | | Not estimable | | |  |
| Cell processing | Primary cells | ≤ 6 | | 20 | | | 8 | | | 5 | | |
|  |  |  |  | 1065 | | | 398 | | | 319 | | |
|  |  |  |  | 0.92 [0.57, 1.48] | | | 0.75 [0.45, 1.25] | | | 0.90 [0.48, 1.71] | | |
|  |  | > 6 to ≤ 12 | | 17 | | | 8 | | | 5 | | |
|  |  |  |  | 876 | | | 357 | | | 150 | | |
|  |  |  |  | 0.66 [0.32, 1.39] | | | 1.14 [0.71, 1.81] | | | 0.75 [0.53, 1.06] | | |
|  |  | > 12 | | 9 | | | 1 | | | 0 | | |
|  |  |  |  | 541 | | | 27 | | | 0 | | |
|  |  |  |  | 0.59 [0.38, 0.91] | | | 0.29 [0.05, 1.62] | | | Not estimable | | |
|  | In vitro cultured cells | ≤ 6 | | 8 | | | 5 | | | 6 | | |
|  |  |  |  | 339 | | | 291 | | | 314 | | |
|  |  |  |  | 0.78 [0.36, 1.71] | | | 1.34 [0.80, 2.25] | | | 1.17 [0.84, 1.63] | | |
|  |  | > 6 to ≤ 12 | | 9 | | | 8 | | | 5 | | |
|  |  |  |  | 668 | | | 484 | | | 610 | | |
|  |  |  |  | 0.94 [0.57, 1.54] | | | 0.74 [0.51, 1.09] | | | 1.06 [0.65, 1.75] | | |
|  |  | > 1 | | 5 | | | 4 | | | 1 | | |
|  |  |  |  | 103 | | | 163 | | | 97 | | |
|  |  |  |  | 0.34 [0.11, 1.01] | | | 0.63 [0.31, 1.29] | | | 1.40 [1.08, 1.82] | | |
| Surgical intervention | Revascularization | ≤ 6 | 8 | | | 6 | | | 4 | | |  |
|  |  |  | 375 | | | 224 | | | 227 | | |  |
|  |  |  | 0.79 [0.21, 3.01] | | | 0.64 [0.16, 2.54] | | | 0.91 [0.40, 2.09] | | |  |
|  |  | > 6 to ≤ 12 | 3 | | | 2 | | | 0 | | |  |
|  |  |  | 156 | | | 99 | | | 0 | | |  |
|  |  |  | 0.47 [0.04, 4.89] | | | 1.13 [0.37, 3.48] | | | Not estimable | | |  |
|  |  | > 12 | 4 | | | 0 | | | 0 | | |  |
|  |  |  | 186 | | | 0 | | | 0 | | |  |
|  |  |  | 1.03 [0.59, 1.78] | | | Not estimable | | | Not estimable | | |  |
|  | No revascularization | ≤ 6 | 15 | | | 5 | | | 1 | | |  |
|  |  |  | 806 | | | 234 | | | 92 | | |  |
|  |  |  | 0.89 [0.56, 1.43] | | | 0.63 [0.42, 0.94] | | | 0.76 [0.30, 1.95] | | |  |
|  |  | > 6 to ≤ 12 | 18 | | | 10 | | | 7 | | |  |
|  |  |  | 1060 | | | 450 | | | 498 | | |  |
|  |  |  | 0.74 [0.45, 1.20] | | | 0.76 [0.54, 1.07] | | | 0.89 [0.57, 1.39] | | |  |
|  |  | > 12 | 8 | | | 3 | | | 0 | | |  |
|  |  |  | 424 | | | 60 | | | 0 | | |  |
|  |  |  | 0.47 [0.32, 0.69] | | | 0.46 [0.22, 0.95] | | | Not estimable | | |  |
| Cell delivery route | CABG+IMI | ≤ 6 | 7 | | 5 | | | 4 | | |  |  |
|  |  |  | 284 | | 194 | | | 197 | | |  |  |
|  |  |  | 1.30 [0.19, 8.88] | | 0.88 [0.17, 4.50] | | | 1.11 [0.60, 2.09] | | |  |  |
|  |  | > 6 to ≤ 12 | 2 | | 1 | | | 0 | | |  |  |
|  |  |  | 96 | | 39 | | | 0 | | |  |  |
|  |  |  | Not estimable | | 1.43 [0.27, 7.61] | | | Not estimable | | |  |  |
|  |  | > 12 | 4 | | 0 | | | 0 | | |  |  |
|  |  |  | 186 | | 0 | | | 0 | | |  |  |
|  |  |  | 1.03 [0.59, 1.78] | | Not estimable | | | Not estimable | | |  |  |
|  | CABG+ICI | ≤ 6 | 2 | | 1 | | | 1 | | |  |  |
|  |  |  | 91 | | 30 | | | 30 | | |  |  |
|  |  |  | 0.95 [0.12, 7.30] | | 0.21 [0.02, 2.07] | | | 0.14 [0.02, 1.20] | | |  |  |
|  |  | > 6 to ≤ 12 | 1 | | 1 | | | 0 | | |  |  |
|  |  |  | 60 | | 60 | | | 0 | | |  |  |
|  |  |  | 0.47 [0.04, 4.89] | | 0.94 [0.20, 4.27] | | | Not estimable | | |  |  |
|  |  | > 12 | 0 | | 3 | | | 0 | | |  |  |
|  |  |  | 0 | | 60 | | | 0 | | |  |  |
|  |  |  | Not estimable | | 0.46 [0.22, 0.95] | | | Not estimable | | |  |  |
|  | TESI | ≤ 6 | 7 | | 4 | | | 1 | | |  |  |
|  |  |  | 399 | | 152 | | | 92 | | |  |  |
|  |  |  | 0.49 [0.21, 1.18] | | 0.52 [0.30, 0.88] | | | 0.76 [0.30, 1.95] | | |  |  |
|  |  | > 6 to ≤ 12 | 8 | | 4 | | | 5 | | |  |  |
|  |  |  | 629 | | 189 | | | 438 | | |  |  |
|  |  |  | 0.54 [0.25, 1.13] | | 0.55 [0.35, 0.86] | | | 0.98 [0.56, 1.70] | | |  |  |
|  |  | > 12 | 6 | | 0 | | | 0 | | |  |  |
|  |  |  | 233 | | 0 | | | 0 | | |  |  |
|  |  |  | 0.29 [0.16, 0.55] | | Not estimable | | | Not estimable | | |  |  |
|  | IMI | ≤ 6 | 2 | | 1 | | | 0 | | |  |  |
|  |  |  | 68 | | 38 | | | 0 | | |  |  |
|  |  |  | 1.77 [0.53, 5.89] | | 1.02 [0.36, 2.88] | | | Not estimable | | |  |  |
|  |  | > 6 to ≤ 12 | 1 | | 0 | | | 0 | | |  |  |
|  |  |  | 30 | | 0 | | | 0 | | |  |  |
|  |  |  | 3.50 [0.50, 24.67] | | Not estimable | | | Not estimable | | |  |  |
|  |  | > 12 | 0 | | 0 | | | 0 | | |  |  |
|  |  |  | 0 | | 0 | | | 0 | | |  |  |
|  |  |  | Not estimable | | Not estimable | | | Not estimable | | |  |  |
|  | ICI | ≤ 6 | 6 | | 1 | | | 0 | | |  |  |
|  |  |  | 347 | | 82 | | | 0 | | |  |  |
|  |  |  | 0.92 [0.41, 2.08] | | 0.82 [0.53, 1.28] | | | Not estimable | | |  |  |
|  |  | > 6 to ≤ 12 | 8 | | 6 | | | 2 | | |  |  |
|  |  |  | 353 | | 253 | | | 60 | | |  |  |
|  |  |  | 0.74 [0.39, 1.41] | | 0.96 [0.59, 1.56] | | | 0.65 [0.26, 1.61] | | |  |  |
|  |  | > 12 | 2 | | 0 | | | 0 | | |  |  |
|  |  |  | 191 | | 0 | | | 0 | | |  |  |
|  |  |  | 0.61 [0.31, 1.19] | | Not estimable | | | Not estimable | | |  |  |
| Delivered cell dose | < 1 mio | ≤ 6 | 1 | | 0 | | | 1 | | |  |  |
|  |  |  | 62 | | 0 | | | 61 | | |  |  |
|  |  |  | 0.48 [0.03, 7.23] | | Not estimable | | | 0.15 [0.03, 0.68] | | |  |  |
|  |  | > 6 to ≤ 12 | 0 | | 0 | | | 0 | | |  |  |
|  |  |  | 0 | | 0 | | | 0 | | |  |  |
|  |  |  | Not estimable | | Not estimable | | | Not estimable | | |  |  |
|  |  | > 12 | 0 | | 0 | | | 0 | | |  |  |
|  |  |  | 0 | | 0 | | | 0 | | |  |  |
|  |  |  | Not estimable | | Not estimable | | | Not estimable | | |  |  |
|  | > 1 to ≤ 10 mio | ≤ 6 | 15 | | 4 | | | 4 | | |  |  |
|  |  |  | 848 | | 284 | | | 258 | | |  |  |
|  |  |  | 0.99 [0.60, 1.63] | | 0.80 [0.53, 1.21] | | | 1.19 [0.81, 1.75] | | |  |  |
|  |  | > 6 to ≤ 12 | 14 | | 6 | | | 3 | | |  |  |
|  |  |  | 735 | | 372 | | | 168 | | |  |  |
|  |  |  | 0.82 [0.39, 1.68] | | 0.78 [0.43, 1.41] | | | 0.68 [0.54, 0.87] | | |  |  |
|  |  | > 12 | 6 | | 0 | | | 0 | | |  |  |
|  |  |  | 365 | | 0 | | | 0 | | |  |  |
|  |  |  | 0.60 [0.34, 1.08] | | Not estimable | | | Not estimable | | |  |  |
|  | > 10 to ≤ 100 mio | ≤ 6 | 5 | | 2 | | | 0 | | |  |  |
|  |  |  | 190 | | 53 | | | 0 | | |  |  |
|  |  |  | 1.15 [0.31, 4.23] | | 1.94 [0.30, 12.66] | | | Not estimable | | |  |  |
|  |  | > 6 to ≤ 12 | 3 | | 4 | | | 2 | | |  |  |
|  |  |  | 99 | | 122 | | | 60 | | |  |  |
|  |  |  | 0.61 [0.17, 2.23] | | 0.93 [0.46, 1.91] | | | 0.65 [0.26, 1.61] | | |  |  |
|  |  | > 12 | 3 | | 2 | | | 0 | | |  |  |
|  |  |  | 79 | | 40 | | | 0 | | |  |  |
|  |  |  | 0.53 [0.16, 1.77] | | 0.48 [0.17, 1.38] | | | Not estimable | | |  |  |
|  | > 100 mio | ≤ 6 | 2 | | 0 | | | 0 | | |  |  |
|  |  |  | 81 | | 0 | | | 0 | | |  |  |
|  |  |  | 0.18 [0.03, 0.97] | | Not estimable | | | Not estimable | | |  |  |
|  |  | > 6 to ≤ 12 | 4 | | 2 | | | 2 | | |  |  |
|  |  |  | 382 | | 55 | | | 270 | | |  |  |
|  |  |  | 0.61 [0.24, 1.55] | | 0.69 [0.26, 1.79] | | | 2.55 [0.54, 12.07] | | |  |  |
|  |  | > 12 | 3 | | 1 | | | 0 | | |  |  |
|  |  |  | 166 | | 20 | | | 0 | | |  |  |
|  |  |  | 0.41 [0.21, 0.80] | | 0.33 [0.10, 1.15] | | | Not estimable | | |  |  |
