# Supplementary material for: Impact of procedural variability and study design quality on the efficacy of cell-based therapies for heart failure - a meta-analysis
Source: PLoS One. 2022 Jan 5;17(1):e0261462. doi: 10.1371/journal.pone.0261462 (PMC8730409; doi:10.1371/journal.pone.0261462)
Supplement: S2 Text — The numbers in brackets indicate the number of studies. (DOCX) [file pone.0261462.s009.docx]

| **Pubmed (31737)** |
| --- |
|  |
| Heart failure AND stem cell transplantation (725) |
| Heart failure AND stem cells (683) |
| Heart failure AND transplantation (5185) |
| Myocardial ischemia AND stem cell transplantation (2809) |
| Myocardial ischemia AND stem cells (3308) |
| Myocardial ischemia AND transplantation (6743) |
| Myocardial infarction AND stem cell transplantation (2133) |
| Myocardial infarction AND stem cells (2395) |
| Myocardial infarction AND transplantation (3414) |
| Cardiomyopathy AND stem cell transplantation (518) |
| Cardiomyopathy AND stem cells (748) |
| Cardiomyopathy AND transplantation (3076) |
|  |
| **Cochrane library (4925)** |
|  |
| Heart failure AND stem cell transplantation (332) |
| Heart failure AND stem cells (516) |
| Heart failure AND transplantation (1494) |
| Myocardial ischemia AND stem cell transplantation (77) |
| Myocardial ischemia AND stem cells (137) |
| Myocardial ischemia AND transplantation (197) |
| Myocardial infarction AND stem cell transplantation (302) |
| Myocardial infarction AND stem cells (531) |
| Myocardial infarction AND transplantation (693) |
| Cardiomyopathy AND stem cell transplantation (121) |
| Cardiomyopathy AND stem cells (195) |
| Cardiomyopathy AND transplantation (330) |
|  |
| **Clincialtrials.gov (842)** |
|  |
| Heart failure AND stem cell transplantation (24) |
| Heart failure AND stem cells (65) |
| Heart failure AND transplantation (172) |
| Myocardial ischemia AND stem cell transplantation (31) |
| Myocardial ischemia AND stem cells (84) |
| Myocardial ischemia AND transplantation (174) |
| Myocardial infarction AND stem cell transplantation (20) |
| Myocardial infarction AND stem cells (82) |
| Myocardial infarction AND transplantation (49) |
| Cardiomyopathy AND stem cell transplantation (18) |
| Cardiomyopathy AND stem cells (43) |
| Cardiomyopathy AND transplantation (80) |
|  |
| **EuDraCT (with results) (76)** |
|  |
| Heart failure AND stem cell transplantation (5) |
| Heart failure AND stem cells (12) |
| Heart failure AND transplantation (38) |
| Myocardial ischemia AND stem cell transplantation (0) |
| Myocardial ischemia AND stem cells (0) |
| Myocardial ischemia AND transplantation (1) |
| Myocardial infarction AND stem cell transplantation (2) |
| Myocardial infarction AND stem cells (6) |
| Myocardial infarction AND transplantation (10) |
| Cardiomyopathy AND stem cell transplantation (0) |
| Cardiomyopathy AND stem cells (0) |
| Cardiomyopathy AND transplantation (2) |
