# Supplementary material for: Impact of procedural variability and study design quality on the efficacy of cell-based therapies for heart failure - a meta-analysis
Source: PLoS One. 2022 Jan 5;17(1):e0261462. doi: 10.1371/journal.pone.0261462 (PMC8730409; doi:10.1371/journal.pone.0261462)
Supplement: Attachment — Submitted filename: Response to Reviewers.docx [file pone.0261462.s010.docx]

Dear Sir and Madam,

thank you for reviewing our manuscript, which we have now revised according to the editor’s and reviewers‘ valuable suggestions. In the following, you find our point-by-point response to the various comments and questions:

**Editors comments:**

1. *Please ensure that your manuscript meets PLOS ONE's style requirements, including those for file naming. The PLOS ONE style templates can be found at*

*https://journals.plos.org/plosone/s/file?id=wjVg/PLOSOne_formatting_sample_main_body.pdf and*

*https://journals.plos.org/plosone/s/file?id=ba62/PLOSOne_formatting_sample_title_authors_affiliations.pdf*

Response: We have applied PLOS ONE’s style requirement throughout.

2*. Thank you for stating the following in the Financial Disclosure Statement Section of your manuscript:*

*"We thank the China Scholarship Council for financial support to XZ, FD and ZL. Funders had no role in study design, data collection and analysis, decision to publish, or preparation of the manuscript."*

*We note that you have provided funding information that is not currently declared in your Funding Statement. However, funding information should not appear in the Acknowledgments section or other areas of your manuscript. We will only publish funding information present in the Funding Statement section of the online submission form. Please remove any funding-related text from the manuscript and let us know how you would like to update your Funding Statement. Currently, your Funding Statement reads as follows: "The authors received no specific funding for this work."*

*Please include your amended statements within your cover letter; we will change the online submission form on your behalf.*

Response: We have removed the funding information from the manuscript text. Please add the following to the Funding Statement section of the online submission system:

“We thank the China Scholarship Council for financial support to XZ, FD and ZL. Funders had no role in study design, data collection and analysis, decision to publish, or preparation of the manuscript."

*3. Please include captions for your Supporting Information files at the end of your manuscript, and update any in-text citations to match accordingly. Please see our Supporting Information guidelines for more information: http://journals.plos.org/plosone/s/supporting-information.*

Response: Captions have been included as requested

4. *We note that this manuscript is a systematic review or meta-analysis; our author guidelines therefore require that you use PRISMA guidance to help improve reporting quality of this type of study. Please upload copies of the completed PRISMA checklist as Supporting Information with a file name “PRISMA checklist”.*

Response: We have uploaded the completed PRISMA checklist

**Reviewers' comments:**

*Reviewer #1: Though the authors have highlighted major findings of the studies involving cell therapy for HF and covered wide range of topics. I believe it would benefit the readers to include a section in the manuscript on future directives summarizing some suggestions on how to have more defined conditions to improve the study design and have more consistent trial procedures; in order to rule out the fact that the non-significant changes seen post transplantation exist due to variations in preparation of cells in the lab or might be the result of inefficiency of the cells by themselves.*

Response: As requested, we have included a paragraph addressing the potential impact of cell product composition in the manuscript. It reads as follows:

“Similarly, autologous cell products have inevitably some inter-individual variability with respect to effector cell dose, number of contaminating cell types, as well as age- and disease-related changes in effector cell biology. Allogenic cell products are more homogenous, at least those derived of a single batch. A more stringent trial design using truly uniform cell products may reduce these confounders but, again, at the cost of more complex cell preparation and quality control measures.”

*Reviewer #2: I recommend the publication of the paper. However, the authors may want to acknowledge that there may have been patient subsets, not taken into account by this study-level meta-analysis, that did benefit from cardiac cell therapy. In this regard, it may be interesting – and provoking – comment the results of this meta-analyses with the echo study of the SHIFT trial: European Heart Journal (2011) 32, 2507–2515*

Response: As requested, we have added a comment on the varying response of individual subsets of patients. It reads as follows:

“This meta-analysis is based on the overall-results of the included trials. In a number of reports, however, attempts were made to identify and characterize patient subpopulations that benefit from cell therapy more than the cohort average suggests. One such phenomenon is that the benefit of the therapeutic intervention inversely correlates with baseline LV function. Obviously, when LV function is only moderately impaired, improvements are more difficult to detect than in patients with decidedly poor baseline contractility. Also, the myocardium may be more amenable to repair in patients with recent onset of disease, compared to thoe with long-standing heart failure and severely scarred/fibrotic myocardium. A machine-learing based post-hoc analysis of patients tertaed with CD133+ BMC in te PERFECT trial revealed that mutations of the SH2B adapter protein 3 (SH2B3), also known as lymphocyte adapter protein (LNK), in hematopietic stem cells predict the outcome of cardiac cell therapy. Hence, it may be that cell therapy has a reproducible effect in selected patients who share the above-mentioned and other characteristics. In the real-world-scenario, however, it would be laborious and impractical to identify such “responder” patients prior to cell therapy.”

As requested, we have also added a comment of the results of the SHIFT trial in relation to the effect of cell therapy:

“Cell therapy must not be an end in itself but a tool for improving symptoms and prognosis of patients with heart failure. As such, it competes with an ever-growing number of established and novel therapeutic interventions. The portfolio of interventions/medications for heart failure is too large to list here but one example is the heart rate reduction by Ivabradine, which inhibits the sodium-potassium inward current (If) that controls spontaneous diastolic depolarization in the sinoatrial node. As shown in the echocardiography substudy of the SHIFT trial, Ivabradine given on top of the standard heart failure medication reversed cardiac remodeling (evidenced by lower LVESDI, higher LVEF and fewer cardiovascular events) to an extent that is very similar to or greater than that seen after cell therapy.”

**Editorial office:**

*While revising your submission, please upload your figure files to the Preflight Analysis and Conversion Engine (PACE) digital diagnostic tool, https://pacev2.apexcovantage.com/. PACE helps ensure that figures meet PLOS requirements. To use PACE, you must first register as a user. Registration is free. Then, login and navigate to the UPLOAD tab, where you will find detailed instructions on how to use the tool. If you encounter any issues or have any questions when using PACE, please email PLOS at figures@plos.org. Please note that Supporting Information files do not need this step.*

Response: We have applied the PACE tool as requested.
